# Substrate-free generation of photosensitizer radical-ion pairs enables multipath photoredox for hypoxia-tolerant photodynamic therapy

**DOI:** 10.1038/s41377-026-02429-9

**Published:** 2026-07-24

**Authors:** Weiyun Yao, Linfang Yang, Ruizhe Chen, Siwei Yao, Haolin Zhang, Mingxuan Jia, Yonghui Pan, Ruida Bai, Zhongzheng Yu, Quli Fan, Wei Huang, Wenbo Hu

**Affiliations:** 1 State Key Laboratory of Flexible Electronics (LoFE), Frontiers Science Center for Flexible Electronics, Institute of Flexible Electronics (IFE), Northwestern Polytechnical University, Xi’an, 710072 China; 2 State Key Laboratory of Flexible Electronics (LoFE) & Institute of Advanced Materials (IAM), Nanjing University of Posts & Telecommunications, Nanjing, 210023 China; 3 Cavendish Laboratory, University of Cambridge, Cambridge, CB3 0HE UK

**Keywords:** Biophotonics, Ultrafast photonics

## Abstract

Photodynamic therapy (PDT) is fundamentally limited by inefficient conversion of photoexcited energy into reactive oxygen species (ROS), especially in hypoxic pathological tissues. Photogenerated radical-ion pairs (PS⁺•/PS⁻•) from photosensitizer could overcome this limitation by broadening photoredox diversity for hypoxia-tolerant ROS production. However, conventional substrate-dependent mechanism typically generates only one radical species, limiting both photoredox diversity and overall ROS production. Here we demonstrate a substrate-free mechanism via noncovalent homodimer fission for efficient generation of PS⁺•/PS⁻• pairs from water-soluble DB-Py, unlocking multipath photoredox for hypoxia-tolerant PDT. Spectroscopic studies and calculations provide experimental evidence for homodimer formation via noncovalent interactions between a photoexcited triplet-state and a ground-state molecule, followed by fission into abundant PS⁺•/PS⁻• pairs. The resulting PS⁺• oxidizes water to generate O_2_ in situ, which is subsequently reduced by PS⁻• to produce •OH and O_2_⁻•, thereby establishing a self‑oxygen‑supplying ROS generation cycle. Consequently, DB-Py achieves 4.9‑fold higher •OH and 2.7‑fold higher O_2_⁻• compared to commercial Rose Bengal, resulting in 7.9-fold enhanced antibacterial efficacy and 2.2-fold accelerated wound epithelialization. This work establishes a substrate-free radical generation mechanism with broad implications for the development of photodynamic, photocatalytic, and photoredox systems.

## Abstract (web summary)

Photoexcited homodimer fission generates PS⁺•/PS⁻• radical pairs that couple water oxidation with oxygen reduction, driving a self-oxygen-supplying ROS cycle for hypoxia-tolerant photodynamic therapy.

## Graphical abstract

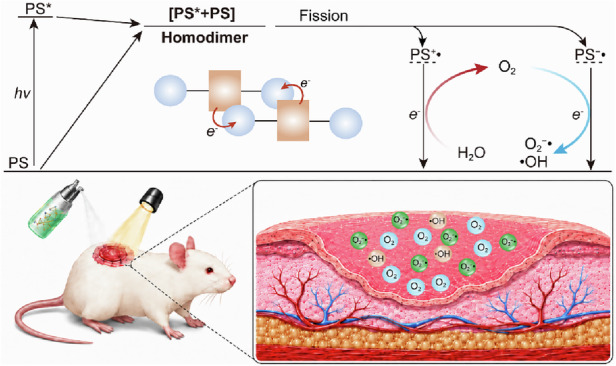

## Introduction

Photogenerated radical-ion pairs (PS⁺•/PS⁻•) derived from photosensitizers (PSs) represent a promising strategy to expand photoredox reactivity by enabling complementary oxidation and reduction processes, thereby underpinning diverse light-driven applications in catalysis, materials science, and biomedicine^1–6^. In photodynamic therapy (PDT), these radical intermediates directly govern the production of cytotoxic reactive oxygen species (ROS) for tumor ablation or antibacterial treatment^6–12^. Specifically, PS⁺• can act as a strong oxidant capable of abstracting electrons from water or biomolecules to generate O_2_ or cytotoxic species, whereas PS⁻• serves as a strong reductant that transfers electrons to O_2_ to form hydroxyl radicals (•OH) or superoxide anions (O_2_⁻•)^13^. When produced simultaneously, PS⁺•/PS⁻• pairs enable multipath photoredox reactivity, markedly broadening ROS diversity and enhancing total ROS output^14^. More importantly, such coupled photoredox processes may establish a self-oxygen-supplying ROS-production cycle to overcome a longstanding limitation of conventional PDT, where ROS generation becomes severely compromised in hypoxic pathological tissues^15–17^. Therefore, developing PSs capable of efficiently generating PS⁺•/PS⁻• pairs is of great importance.

**Fig. 1:**
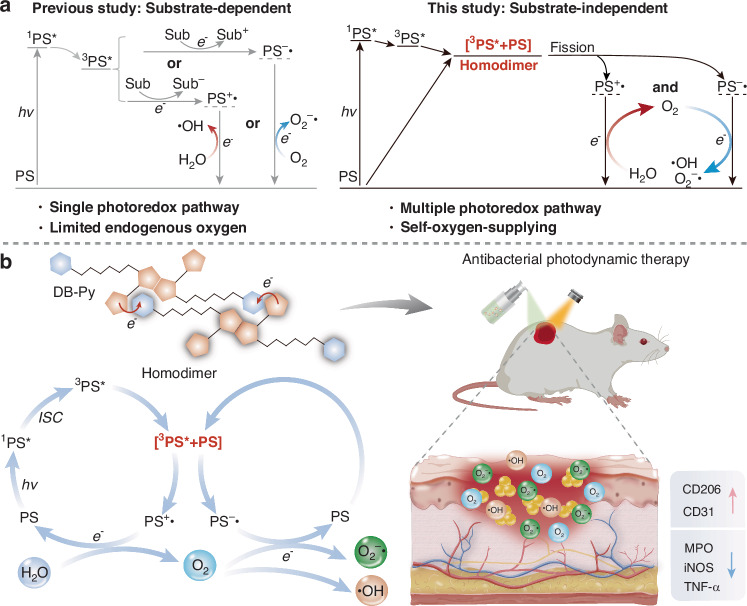
Substrate-free generation of photosensitizer radical-ion pairs via noncovalent homodimer fission for hypoxia-tolerant photodynamic therapy. **a** Substrate-independent and substrate-dependent mechanisms for photosensitizer radical-ion generation and the corresponding photoredox processes. **b** Schematic illustration of noncovalent homodimerization and the self-oxygen-supplying ROS generation cycle by homodimer fission for antibacterial photodynamic therapy

The efficient generation of PS⁺•/PS⁻• pair remains challenging because direct experimental demonstration of their formation is still limited and reliable design principles remain underdeveloped^18–20^. Current strategies predominantly rely on substrate-dependent intermolecular electron transfer (*Inter*ET) between PSs and external electron-donating or -withdrawing substrates (Fig. 1a), which usually produces only one type of PS radical ion^21,22^. This confines photoredox activity to a single pathway, thereby limiting ROS diversity and overall ROS yield. Moreover, the reliance on external redox substrates increases system complexity and compromises reproducibility, thus hindering clinical translation^23–26^. A more appealing alternative is substrate-free *Inter*ET between identical PS molecules, which could directly generate PS⁺•/PS⁻• pairs in a simplified system^27,28^. Homodimeric PSs with accessible triplet excited states (^3^PS*) are particularly promising for this strategy, as long-lived ^3^PS* are much more likely to undergo productive *Inter*ET than short-lived singlet states (^1^PS*)^29^. Diketopyrrolopyrrole (DPP) is well suited to this design because it combines an accessible ^3^PS* manifold with an intrinsic tendency to form homodimers over a broad concentration range^30^. However, its potential for substrate-free PS⁺•/PS⁻• pairs generation has not yet been explored.

Here, we report a DPP-based PS (DB-Py) that undergoes noncovalent homodimer fission to generate abundant PS⁺•/PS⁻• pairs, enabling hypoxia-tolerant ROS production for superior PDT (Fig. 1). Spectroscopic analyses confirm stable homodimer formation of DB-Py. Nuclear Overhauser Effect Spectroscopy (NOESY) and calculations reveal preferential intermolecular coupling between the side-chain pyridine acceptor and the adjacent DPP backbone donor, which stabilizes the homodimer. Femtosecond transient absorption (fs-TA) spectroscopy combined with spectroelectrochemical (SEC) analysis further shows that this packing mode promotes both high ^3^PS* population for single oxygen (^1^O_2_) generation and homodimer formation between ^3^PS* and ground-state PS. The homodimer fission via *Inter*ET promotes photoreduction at the pyridine acceptor to form DB-Py⁻• and photooxidation at the DPP donor to generate DB⁺•-Py, which subsequently engage in downstream photoredox reaction to enable self-oxygen-supplying Type I ROS production. Therefore, DB-Py achieves a 2.7-fold increase in O_2_⁻• and a 4.9-fold increase in •OH production compared to commercial Rose Bengal (RB), affording a 7.9-fold increase in antibacterial activity and 2.2-fold acceleration in epithelialization in full-thickness wound models.

## Results

### Molecular design and spectroscopic properties

To elucidate structure-property relationships, three DPP derivatives (D-Py, DB-TE, and DB-Py) were designed via side-chain modifications and bromination. Water-soluble pyridinium and triethylammonium cations were introduced to improve aqueous solubility and promote intermolecular interactions. Bromination was initially designed to enhance intersystem crossing (ISC), but was later found to promote noncovalent homodimerization by enhancing intermolecular interactions. Detailed synthetic procedures and structural characterization, including ^1^H NMR,^13^C NMR and HPLC-MS analyses, are provided in Supplementary Scheme 1–6 and Fig. S1–10.

**Fig. 2:**
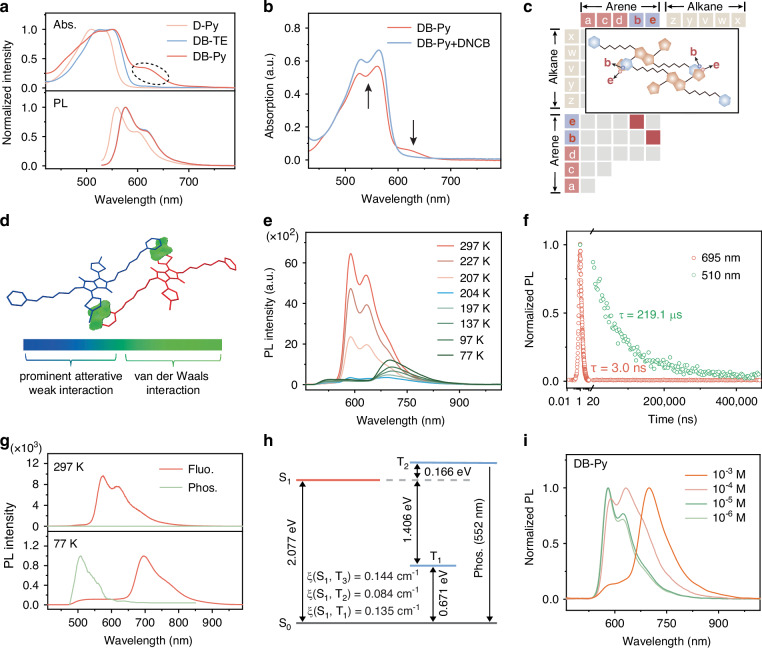
Experimental evidence for homodimer formation. **a** Absorption and PL spectra in aqueous solution. **b** Absorption spectra of DB-Py in DMSO/H_2_O mixed solvent with and without the excited-state quencher DNCB. **c** Diagram of NOESY spectrum of DB-Py. **d** Weak interaction analysis of DB-Py homodimer with independent gradient model based on Hirshfeld partition (IGMH). **e** Temperature-dependent PL spectra of DB-Py. **f** Time-resolved PL spectrum of DB-Py in H_2_O monitored at 695 nm (λ_*ex*_: 532 nm, 80.3 ps, 1 kHz) and 510 nm at 77 K (λ_*ex*_: 355 nm, 80.3 ps, 500 Hz). **g** Fluorescence and phosphorescence (1 ms delayed) spectra of DB-Py recorded at 297 K and 77 K. **h** Calculated ISC channels from S_1_ state to triplet state for DB-Py. **i** Concentration-dependent PL spectra of DB-Py in H_2_O

All three PSs in aqueous solution exhibit strong absorption with molar extinction coefficients on the order of 10^4 ^M^–1^ cm^–1^ (Fig. 2a and Fig. S11), approximately one order of magnitude higher than that of the commercial PS porphyrin (1.2 × 10^3 ^M^–1^ cm^–1^)^31^. DB-TE and DB-Py display red-shifted absorption and photoluminescence (PL) maximum, attributed to the introduction of bromine atom on the thiophene unit. The PL quantum yield (PLQY) of D-Py reaches 31.3%, while DB-TE and DB-Py show lower PLQYs of 19.7% and 18.2%, respectively. This relatively low PLQY of DB-Py indicates substantial nonradiative dissipation of photoexcited energy, potentially involving ISC, heat release, and structural/vibrational relaxation.

Comparison of the photothermal response and Stokes shift suggest that nonradiative dissipation in DB-Py is mainly channeled through ISC rather than heat generation or structural/vibrational relaxation. All three PSs induced only slight temperature increases, with DB-Py showing the weakest photothermal response (Fig. S12), indicating very limited heat-generating nonradiative decay. DB-Py also exhibited the smallest Stokes shift, suggesting suppressed structural/vibrational relaxation (Table. S1). In addition, DB-Py shows a shorter PL lifetime of 3.1 ns than DB-TE (3.2 ns) and D-Py (4.1 ns) (Fig. S13). This shortened lifetime is likely a consequence of bromination-enhanced spin-orbit coupling, which facilitates ISC from lowest singlet state (S_1_) to ^3^PS*. Together, these results indicate that DB-Py minimizes energy loss through heat dissipation and structural/vibrational relaxation, thereby allowing more photoexcited energy to drive ^3^PS* formation and subsequent ROS generation.

### Spontaneous formation of homodimer

A pronounced absorption peak around 615 nm, observed exclusively for DB-Py (Fig. 2a), provides experimental evidence for homodimer formation^32^. The marked attenuation of this peak upon the addition of the excited-state quencher 2,4-dinitrochlorobenzene (DNCB) suggests that the 615 nm absorption primarily arises from excited-state absorption (ESA), rather than from ground-state (S_0_) transitions (Fig. 2b)^33^. Nuclear Overhauser Effect Spectroscopy (NOESY) analysis reveals distinct cross-peaks between the pyridinic proton on the side chain and the protons on the DPP conjugated backbone of neighboring DB-Py molecules (Fig. 2c and Fig. S14), demonstrating an intermolecular head-to-side stacking mode. In this configuration, the sidechain pyridine lies in close spatial proximity to the DPP core of an adjacent molecule. Consistent with this experimental observation, conformational searching also identifies such head-to-side packed dimers as energetically favorable configuration for DB-Py (Fig. S15 and Table S2). Weak interaction analysis further reveals extensive van der Waals interaction between side-chain pyridine and the DPP backbone, as evidenced by the broad green isosurfaces (Fig. 2d). In addition, electrostatic potential mapping reveals that bromination markedly strengthens the intermolecular electrostatic interaction between the cationic pyridine moiety and the adjacent DPP backbone by lowering the local potential of the thiophene fragment from 86 to 42 kcal/mol (Fig. S16), thereby stabilizing the head-to-side stacked homodimer.

### Nature of homodimer

Spectroscopic evidence reveals that DB-Py homodimer formation promotes ISC and enables efficient population of ^3^PS*. At room temperature (297 K), the PL spectrum displays a distinct emission peak near 575/617 nm (Fig. 2e). As the temperature decreases to 77 K, these emissions are progressively suppressed, while two new bands at ~695 nm and ~510 emerged and became increasingly pronounced. This temperature-dependent spectral evolution likely arises from the promoted singlet-to-triplet ISC at low temperature, which suppresses singlet-involved emission at 575/617 nm while increasing triplet-involved emission at ~695 and ~510 nm. Time-resolved PL measurements at 77 K assign the 510 nm emission to phosphorescence because of its long lifetime of 219.1 μs (Fig. 2f), whereas the 695 nm band exhibits a short lifetime of 3.0 ns. This assignment is corroborated by the delayed PL spectrum recorded with a 1 ms delay (Fig. 2g), where the 695 nm emission disappears while the 510 nm phosphorescence remains clearly visible.

Theoretical calculations based on the optimized homodimer structure assign the 510 nm phosphorescence to the higher triplet excited state (T_2_), rather than the lowest triplet state (T_1_) as traditionally assumed from Kasha’s rule. The calculated T_2_–S_0_ energy gap matches well with the experimental phosphorescence maximum (Fig. 2h and Fig. S17), validating the reliability of computational model. This calculation also reveals a reduced S_1_ → T_2_ energy gap and a markedly enhanced spin–orbit coupling of the dimerized DB-Py monomer relative to the isolated monomer (Fig. S18), likely owing to its more twisted conjugated framework. These features facilitate S_1_ → T_2_ ISC and thus generate a substantial population of T_2_ states (Fig. 2f). Moreover, the large T_2_–T_1_ energy separation suppresses T_2_ → T_1_ internal conversion and rationalizes the unusual T_2_ → S_0_ phosphorescence.

DB-Py homodimer formation is further proposed to be initiated by the collision of one photoexcited ^3^PS* and a ground-state molecule. Since higher concentration favors intermolecular collisions, we performed concentration-dependent PL measurements. As the DB-Py concentration increased, the 695 nm emission band became progressively stronger (Fig. 2i), supporting its assignment to homodimer emission. The involvement of ^3^PS* in this homodimer-associated emission was further verified by vitamin E (VE), a commonly used ^3^PS* quencher^34^. As shown in Fig. S19, the 695 nm emission gradually decreased with increasing VE concentration, providing strong evidence for ^3^PS* participation in DB-Py homodimer formation and emission. Together, the close intermolecular packing within the homodimer and the substantial population of long-lived T_2_ states provide a reasonable basis for increased collisions between T_2_-state and neighboring S_0_ molecule, thereby facilitating T_2_–S_0_ homodimer formation. Notably, the emergence of a 695 nm band in all three PSs indicates that homodimer formation is a general feature of this molecular system (Figs. S20–21), with DB-Py showing the strongest dimerization tendency.

### Experimental observation of PS^+^•/PS^−^• pairs generation

**Fig. 3:**
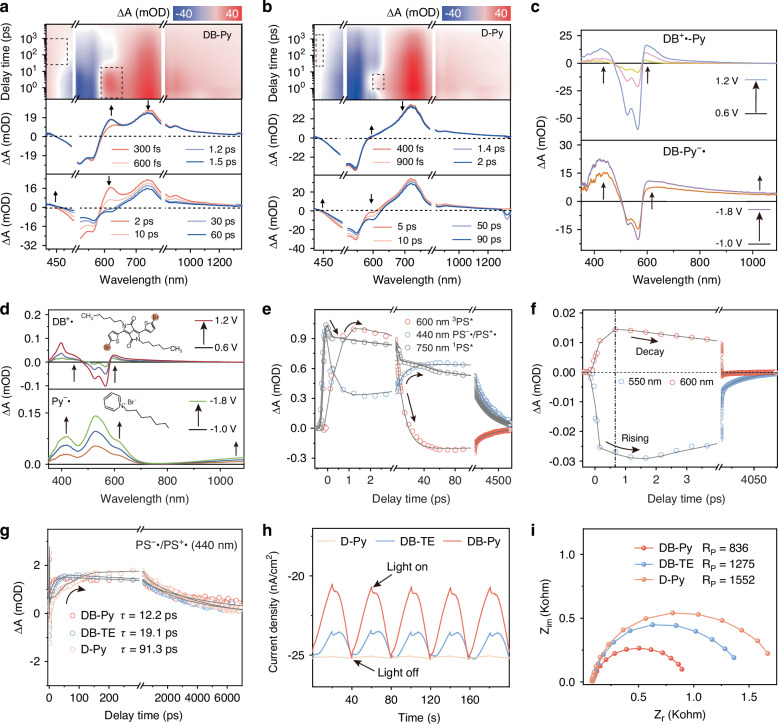
Experimental evidence for photogenerated PS^+^•/PS^−^• pair. **a**, **b** fs-TA mapping of DB-Py and D-Py in aqueous solution pump at 500 nm. Extracted fs-TA plots under different delay time (below). The up and down arrow indicate buildup and decay of species, respectively. **c** Spectroelectrochemical characterization of DB-Py in DCM under different applying biases. **d** Spectroelectrochemical characterization of DPP backbone (DB) and pyridine (Py) segment. **e** Kinetics traces for DB-Py at the representative wavelength within triplet (600 nm), radical (440 nm) and singlet (750 nm) ESA region. **f** Kinetic traces for DB-Py at the wavelength within triplet ESA (600 nm) and GSB (550 nm) region. **g** Kinetic traces of DB-Py, DB-TE and D-Py at 440 nm corresponding to the ESA region of PS^+^• and PS⁻•. **h** Representative photocurrent responses of DB-Py, DB-TE and D-Py. **i** Charge-transfer resistance of DB-Py, DB-TE and D-Py under photoirradiation

Femtosecond transient absorption (fs-TA), combined with spectroelectrochemical (SEC) characterization, provides direct experimental observation for PS⁺•/PS⁻• pairs generation. The fs-TA spectra of DB-Py, DB-TE, and D-Py (Fig. 3a, b and Fig. S22) display prominent ground-state bleaching (GSB) around 550 nm together with three ESA bands centered at ~440, 600, and 750 nm. The 750 nm ESA band is assigned to the singlet excited state because its decay time matches well with the fluorescence lifetime (Fig. S23). Notably, its decay is accompanied by sequential rise of the 600 and 440 nm ESA bands (Fig. 3a), revealing the stepwise formation of distinct transient species. Specifically, at early delay times, the 600 nm band rises as the 750 nm band decays. At longer delay times, the 600 nm band gradually decreases while the 440 nm band continues to grow, suggesting further conversion into another transient species.

SEC analysis was then used to identify the characteristic spectral signatures of PS⁺•/PS⁻• species by applying controlled positive and negative biases to selectively generate the corresponding radical species. Electrochemical measurements provide suitable positive and negative biases for the three PSs (Fig. S24). Increasing the positive bias from 0.6 to 1.2 V generated new bands at ~440 and 600 nm, which are assigned to PS⁺• (Fig. 3c). Conversely, negative biases from −1.0 to −1.8 V induced broad absorption around 440 nm and above ~600 nm, attributable to PS⁻• (Fig. 3c). The ~440 and ~600 nm ESA bands in fs-TA match well with the characteristic radical absorptions observed for DB-Py in SEC, and are therefore assigned to photogenerated PS⁺•/PS⁻• pairs. In contrast, D-Py and DB-TE show very weak radical-associated absorptions in either the fs-TA or SEC spectra (Fig. S25), indicating inefficient PS⁺• or PS⁻• formation. These results not only provide direct experimental evidence for photogenerated PS⁺•/PS⁻• pairs, but also establish a rigorous methodology for the characterization of photogenerated radical species.

Further fragment-based SEC analysis of the pyridine (Py) and DPP backbone (DB) assigns the radical species in DB-Py to DB⁺•-Py and DB-Py⁻• (Fig. 3d), suggesting that the photoinduced charges are localized on distinct molecular segments rather than delocalized across the entire conjugated framework. Specifically, oxidation of the DB fragment generates absorption bands at ~440 and 600 nm, which are assigned to DB⁺• (Fig. 3d). In parallel, reduction of the Py fragment produces absorption features at ~440 and 600 nm, together with a broad band around ~800 nm, corresponding to Py⁻•.

### Excited-state dynamics for generating PS^+^•/PS^−^• pairs

The stepwise growth of the 600 and 440 nm ESA bands is primarily assigned to ^3^PS* and PS⁺•/PS⁻• pairs, respectively (Fig. 3e). Together with strong phosphorescence (Fig. 2e) and theoretical predictions of facile ISC (Fig. 2h), fs-TA results support a major contribution of ^3^PS* to the 600 nm ESA band. The rise of the 600 nm ESA band is markedly faster in DB-Py (726 fs) and DB-TE (735 fs) than in D-Py (994 fs), indicating the most efficient ^3^PS* formation in brominated systems, particularly in DB-Py (Fig. S26). Notably, DB-Py exhibits a rising GSB signal during the decay of the 600 nm ^3^PS* ESA band, corresponding to depletion of S_0_ likely induced by T_2_-S_0_ collisions for homodimer formation (Fig. 3f). This behavior indicates that ^3^PS* participates in the generation of PS⁺•/PS⁻• pairs and supports assignment of the 440 nm ESA band to the radical-ion pairs, thus accounting for its delayed appearance relative to the 600 nm ESA band. The thermodynamic feasibility of fission of T_2_–S_0_ homodimer into PS⁺•/PS⁻• pairs was further demonstrated from the vertical ionization potential (VIP) and vertical electron affinity (VEA) of the PSs. The calculations show that this fission is energetically allowed only between one T_2_ and one neighboring S_0_ molecule (Table S3). Together, these results support the conclusion that the PS⁺•/PS⁻• pairs originate from fission of the T_2_–S_0_ homodimer.

Kinetic analysis at 440 nm further reveals faster formation and a much longer lifetime of the PS⁺•/PS⁻• pair in DB-Py than in DB-TE and D-Py (Fig. 3g). The radical species in DB-Py form within 12.2 ps and persist for 762 ps, whereas those in DB-TE and D-Py form more slowly (19.1 and 91.3 ps) and decay much faster (253 and 193 ps). Because long-lived radical ions generally exhibit enhanced reactivity towards surrounding substrates, these dynamics are expected to favor more efficient ROS generation. The relative radical yields, estimated from the ratio of corresponding intensity changes between the 440 nm ESA and 550 nm GSB signals, follow the order DB-Py (25.0%) > DB-TE (15.1%) > D-Py (12.9%) (Fig. S27). This trend is in good agreement with the much higher photocurrent response of DB-Py relative to D-Py and DB-TE (Fig. 3h), indicating more efficient charge separation. Electrochemical impedance spectroscopy likewise reveals a much lower charge-transfer resistance for DB-Py (Fig. 3i), suggesting kinetically favored charge separation that suppresses charge recombination. These results demonstrate that the DB-Py homodimer combines efficient ISC with facile fission, thereby enabling substantial formation of long-lived PS⁺•/PS⁻• pairs.

### Multipath photoredox for hypoxia-tolerant ROS production

**Fig. 4:**
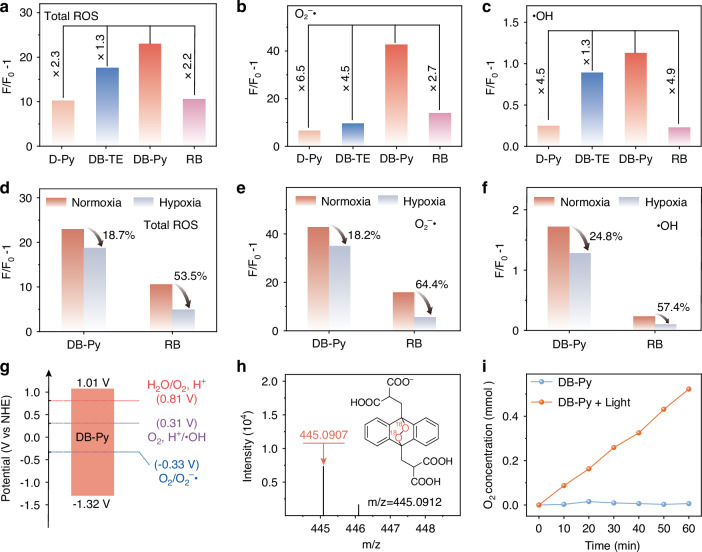
Experiment evidence for ROS production. **a–c** Total ROS, O_2_⁻• and •OH generation of DB-Py compared to D-Py, DB-TE and RB. **d**–**f** Total ROS, O_2_⁻• and •OH generation of DB-Py and RB under normoxia and hypoxia. **g** Redox potentials diagram of DB-Py. **h** Isotopic mass spectrometry spectra of photoinduced ^18^O_2_ production from DB-Py in H_2_^18^O solution after 30 min white-light irradiation. **i** O_2_ evolution curves of DB-Py with or without white-light irradiation

Substantial formation of PS⁺•/PS⁻• pairs endows DB-Py with significantly enhanced •OH and O_2‍_**⁻**‍• generation relative to D-Py and DB-TE. Total ROS, ^1^O_2_, O_2_**⁻**• and •OH production were evaluated using 2′,7′-dichlorodiydrofluorescein (DCFH), 2,2′-(anthracene-9,10-diylbis (methylene)) dimalonic acid (ABDA), dihydrorhodamine 123 (DHR 123) and aminophenyl fluorescein (APF), respectively. Rose Bengal (RB), a commercial PS with compatible excitation wavelength, was used as a benchmark. Under identical conditions, DB-Py generated 2.3- and 1.3-fold higher total ROS in aqueous solution than D-Py and DB-TE, respectively (Fig. 4a and Fig. S28). In addition, its total ROS output was 2.2 times that of RB, underscoring its superior photodynamic effect. More strikingly, DB-Py displayed greatly enhanced Type I ROS generation, with O_2_**⁻**• production increased by 6.5-fold and 4.5-fold relative to D-Py and DB-TE, and •OH production increased by 4.5-fold and 1.3-fold, respectively (Fig. 4b, c and Fig. S29–30). It also surpassed RB by 2.7-fold in O_2_**⁻**• generation and 4.9-fold in •OH generation. In addition, DB-Py generated more ^1^O_2_ than D-Py and DB-TE (Fig. S31), despite lower ^1^O_2_ output than RB. Notably, under hypoxia, DB-Py showed only ~20% decreases in total ROS, O_2_⁻• and •OH generation, whereas RB lost more than half of its activity (Fig. 4d–f and Fig. S32). Together, these results demonstrate DB-Py as a hypoxia-tolerant PS with strong promise for PDT even in hypoxic pathological environments.

### Mechanisms for hypoxia-tolerant ROS production

To gain mechanistic insight into hypoxia-tolerant ROS production, we examined the redox properties of DB-Py. DB-Py exhibits an oxidation potential of 1.0 V and a reduction potential of −1.3 V versus NHE (Fig. 4g). The strongly negative reduction potential thermodynamically permits electron transfer from DB-Py to O_2_, as it is more negative than the redox potentials of both O_2_/H^+^/•OH (0.31 V vs. NHE, pH = 7) and O_2_/O_2_⁻• ( − 0.33 V vs. NHE, pH = 7). This energetic alignment supports the efficient formation of O_2_⁻• and •OH, consistent with the experimentally observed robust Type I ROS generation (Fig. 4b, c). Meanwhile, the more positive oxidation potential relative to the H_2_O/O_2_ (0.81 V vs. NHE) indicates that DB-Py^+^• can drive water oxidation to generate O_2_ in situ, thereby helping sustain ROS production under hypoxic conditions.

This water oxidation-driven O_2_ evolution was further confirmed by isotopic mass spectrometry and dissolved-oxygen measurements. When H_2_^18^O was used as the water source, illumination of DB-Py led to formation of ^18^O_2_, which was subsequently converted into ^1^O_2_ and trapped by ABDA to form the corresponding endoperoxide. A characteristic signal at m/z = 445.0907 was detected only in the DB-Py/ H_2_^18^O system (Fig. 4h), but not in the H_2_^16^O control (Fig. S33). Dissolved-oxygen measurements further revealed a light-induced O_2_ evolution rate of 0.5 mmol h^−1^ g^−1^ (Fig. 4i). These results experimentally demonstrate that DB-Py can oxidize water to generate O_2_ in situ, supporting sustained ROS production under hypoxia.

These results support the proposed mechanistic scheme (Fig. 1). Upon photoexcitation, DB-Py undergoes S_1_ → T_2_ ISC, producing a substantial T_2_ population. These long-lived T_2_ molecule preferentially couple with neighboring S_0_ molecules to form homodimers, which subsequently undergoes fission into DB⁺•-Py and DB-Py⁻•. DB⁺•-Py oxidizes water to generate O_2_ in situ, whereas DB-Py⁻• reduces endogenous or newly generated O_2_ to yield O_2_⁻• and •OH. In parallel a fraction of the T_2_ population can also transfer energy to O_2_ to produce ^1^O_2_. The predominance of Type I ROS over ^1^O_2_ likely stems from the preferential consumption of the T_2_ state through homodimer formation, which outcompetes energy transfer to O_2_ and thereby suppresses ^1^O_2_ generation.

### In vitro and in vivo antibacterial activity

**Fig. 5:**
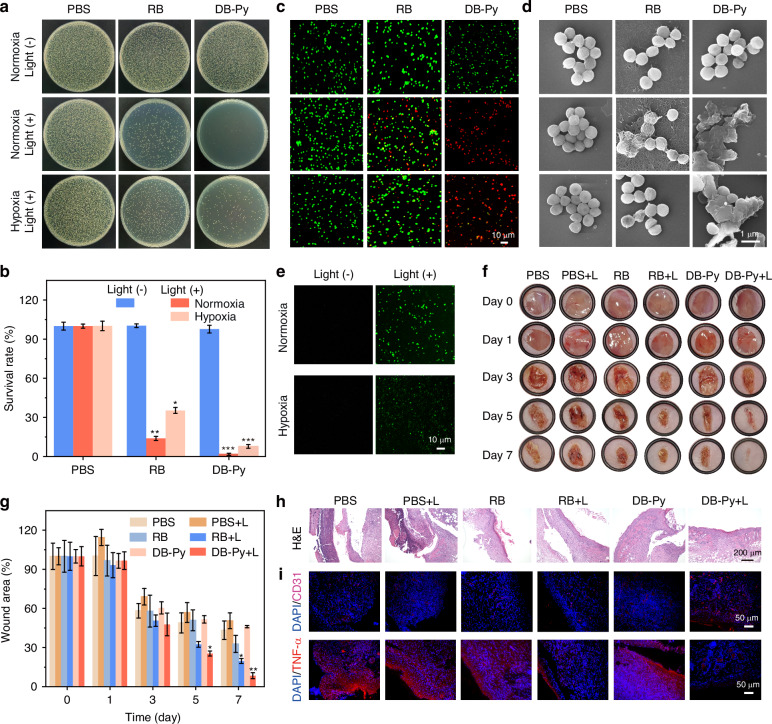
In vitro and in vivo antibacterial effects of DB-Py. **a** Comparison of antibacterial plate images between RB and DB-Py under normoxia and hypoxia. **b** Survival rate of MRSA with RB and DB-Py under normoxia and hypoxia (data presented mean ± SD, *n* = 3). **c** Fluorescence images of the live/dead stained bacteria under normoxia and hypoxia. **d** SEM images of MRSA after treatment with different samples under normoxic and hypoxic conditions. **e** ROS generation in MRSA under white-light irradiation in normoxic and hypoxic conditions. **f** Representative photographs of MRSA-infected wounds in mice after different treatments. **g** Quantitative analysis of wound area. (data presented mean ± SD, *n* = 4). **h** H&E-stained images of wound tissue after 8 days of treatment. **i** Immunohistochemical staining of CD31 and TNF-α in wound tissues from different groups. *p < 0.05, **p < 0.01, ***p < 0.001

The antibacterial activity of DB-Py was evaluated against methicillin-resistant *Staphylococcus aureus* (MRSA) under normoxic (21% O_2_) and hypoxic ( < 1% O_2_) conditions. Plate counting assays demonstrated significant antibacterial activity of DB-Py under white-light irradiation (λ > 495 nm, 50 mW cm^-2^), while negligible antibacterial effects were observed without light (Fig. 5a). Notably, quantitative analysis revealed a bacterial inhibition rate of 98.3% (*p* < 0.001 vs control), representing a 7.9-fold enhancement relative to RB (Fig. 5b). Importantly, DB-Py maintained high antibacterial efficacy under both normoxic (98.3%) and hypoxic (92.3%) conditions, whereas RB showed a marked decline from 86.1% to 74.6% (Fig. 5a, b), highlighting the strong hypoxia tolerance of DB-Py. Live/dead staining and scanning electron microscopy (SEM) further supported this conclusion. SYTO 9 and propidium iodide (PI) were employed to identify live and dead bacteria, emitting green and red fluorescence, respectively. MRSA treated with PBS or light alone showed predominantly green fluorescence, whereas DB-Py under irradiation induced strong red fluorescence under both normoxic and hypoxic conditions, indicating extensive bacterial death (Fig. 5c). SEM images likewise showed severe membrane shrinkage and collapse in DB-Py-treated bacteria after irradiation, while non-irradiated groups retained intact morphology (Fig. 5d). DCFH-DA staining further confirmed robust intracellular ROS generation in MRSA cells treated with DB-Py under irradiation in both oxygen conditions (Fig. 5e). Together, these results demonstrate the excellent in vitro antibacterial performance of DB-Py.

Encouraged by the in vitro antibacterial activity, we next evaluated the antibacterial efficacy of DB-Py in vivo using an MRSA-infected dorsal wound model in mice. Wound progression was photographed every two days after infection, and body weight was monitored throughout treatment (Fig. S34). Quantitative wound analysis revealed that the DB-Py + L group achieved 91.6% wound closure and a 2.2-fold acceleration in re-epithelialization relative to the RB group (Fig. 5f, g). On day 8, mice were sacrificed and wound tissues were collected for histological evaluation using haematoxylin and eosin (H&E) and Masson’s trichrome staining. H&E staining showed that the DB-Py + L group exhibited minimal neutrophil infiltration and more preserved skin architecture (Fig. 5h), in sharp contrast to the pronounced inflammation and tissue disruption observed in the other groups. Masson staining further revealed enhanced collagen deposition and improved tissue integrity in the DB-Py + L group, consistent with accelerated wound healing (Fig. S35). These findings confirm strong in vivo antibacterial activity of DB-Py.

To further evaluate wound healing, we performed immunofluorescence staining on the wound tissues. CD31, a marker of angiogenesis and tissue regeneration^35^, showed a strong fluorescence signal in the DB-Py + L group (Fig. 5i), indicating active neovascularization. Notably, this group also exhibited lower TNF-α expression, suggesting effective suppression of the inflammatory response. Myeloperoxidase (MPO) and inducible nitric oxide synthase (iNOS), both associated with inflammatory activation^36,37^, were barely detectable in the DB-Py + L group compared with the control group (Fig. S36), indicating a relatively mild inflammatory state. Furthermore, CD206, recognized as a marker for anti-inflammatory or reparative M2-like macrophages, showed pronounced expression in the DB-Py + L group, suggesting that inflammation had entered the resolution phase (Fig S36). These results collectively confirm that DB-Py effectively alleviates wound inflammation, promotes angiogenesis and tissue regeneration, and thereby accelerates the healing of infected wounds.

### Biosafety

The biosafety of DB-Py was evaluated through standard hematological, biochemical, and histological analyses. In vitro, DB-Py showed excellent cytocompatibility towards NIH 3T3 fibroblasts, with high cell viability maintained after 24 and 48 h of co-incubation (Fig. S37). Moreover, DB-Py exhibited no detectable spectral changes after 7 days of storage in a simulated physiological environment containing 10% FBS (Fig. S38), underscoring its optical robustness. In vivo, no obvious body-weight changes were observed in any treatment group (Fig. S39), indicating that DB-Py suppresses infection without causing systemic toxicity. Histological examination of major organs, including the heart, liver, spleen, lungs, and kidneys, revealed no discernible abnormalities or lesions (Fig. S40). In vivo metabolic pathway analysis further showed no detectable fluorescence signals in major organs within 24 h post-administration (Fig. S41). In addition, hematological and biochemical analyses showed no significant differences in blood parameters among the groups (Figs. S42–43), indicating negligible hepatotoxicity, nephrotoxicity, and hematological toxicity. These results collectively confirm the excellent in vivo biosafety of DB-Py.

## Discussion

This study establishes a substrate-free mechanism for generating long-lived PS⁺•/PS⁻• pairs through noncovalent homodimer fission, enabling hypoxia-tolerant ROS production for highly effective antibacterial PDT. The resulting PS⁺•/PS⁻• pairs couple water oxidation with oxygen reduction to create a self-oxygen-supplying ROS production cycle, providing an innovative strategy to overcome the longstanding oxygen limitation of conventional PDT. Mechanistically, homodimer plays a central role in redirecting photon energy towards productive ROS generation. Methodologically, the integration of fs-TA, SEC, and theoretical calculations establishes a broadly applicable framework for the rigorous identification of photogenerated radical intermediates and the mechanistic elucidation of their evolution, thus enabling the rational control of radical photoredox processes. From a translational perspective, the substrate-free design affords a single-component PS platform with reduced complexity and improved reproducibility, enhancing its promise for clinical translation. Overall, this work deepens the mechanistic understanding of radical photoredox in photodynamic systems and establishes a versatile framework for the mechanistic interrogation and rational design of next-generation radical-mediated systems for biomedicine and beyond.

## Materials and methods

All reagents are commercially available and used directly without purification. Experimental protocols include material synthesis, photocurrent responses, charge-transfer resistance, isotopic mass spectrometry, dissolved-oxygen, in vitro and in vivo antibacterial experiments, as well as the details of experimental instruments are described in supplementary information.

### Spectroscopic measurement

Steady-state absorption spectra were acquired using a double-beam UV–Vis–NIR spectrophotometer (Hitachi UH-5300, Japan). Steady-state and time-resolved PL spectra were recorded using a fluorospectrometer (FF4-SL, Orient KOJI Ltd., China). The absolute PLQY was measured with a Quantaurus-QY Plus system (Hamamatsu C13534-31). In addition, fs-TA measurements were carried out on a home-built setup configured according to our previously reported method^38^.

### Spectroelectrochemical measurements

Spectroelectrochemical measurements were performed using a home-built setup comprising an electrochemical workstation, a halogen light source, and an optical fiber spectrometer. An AUTOLAB electrochemical analyzer was operated in a standard three-electrode configuration, with a platinum grid as the counter electrode, an Ag/AgCl electrode as the reference electrode, and a platinum rod as the working electrode. The electrodes were placed in a 1 mm cuvette containing a solution of 10 mM tetrabutylammonium hexafluorophosphate ([Bu_4_N] [PF_6_]) in DCM as the supporting electrolyte. The cuvette holder was equipped with optical entry and exit ports, allowing light to pass through the sample solution. Light from a halogen lamp (HL-2000-LL, Ocean Insight) was focused into the cuvette, and the transmitted signal was collected by an optical fiber spectrometer (PG2000-Pro back-thinned spectrometer, Ideaoptics, China). The spectrometer and electrochemical controller were synchronized using home-built software, enabling simultaneous electrochemical modulation and spectral recording.

### Theoretical calculations

Ground-state geometries were fully optimized using density functional theory (DFT) with the M06-2X-D3 functional and 6-31 G (d, p) basis set. Excited-state optimizations for the lowest singlet S_1_ and triplet T_1_ states were performed using time-dependent DFT (TD-DFT) with the same functional and basis set. All optimized structures were verified via harmonic vibrational frequency calculations. These calculations were conducted using the Gaussian 16 A.03 program^39^. Spin-orbit coupling matrix elements (SOCME) and excitation energies were calculated using the ORCA 5.0.3 program^40^, employing the TD-revPBE0 functional with the Def2-TZVP basis set. Vertical ionization potentials (VIP) and vertical electron affinity (VEA) were calculated using with M06-2X-D3 functional and 6-311 + G (d, p) basis set. Total energies of the neutral (ground state), anion radical, and cation radical PS molecules were computed via DFT. The S_1_ were evaluated using time-dependent DFT (TD-DFT), and the T_1_ states were calculated using unrestricted DFT (UDFT). The SMD solvent model was applied to simulate a water environment.

## Supplementary information

Supplementary Information

## Data Availability

All data supporting the findings of this study are available within the paper and its supplementary materials. Additional data are available from the corresponding author upon reasonable request.
