# Supplementary material for: Substrate-free generation of photosensitizer radical-ion pairs enables multipath photoredox for hypoxia-tolerant photodynamic therapy
Source: Light Sci Appl. 2026 Jul 24;15:331. doi: 10.1038/s41377-026-02429-9 (PMC13400614; doi:10.1038/s41377-026-02429-9)
Supplement: Supplementary file 1 — Supplementary Information [file 41377_2026_2429_MOESM1_ESM.pdf]

Supplementary Information for

Substrate-Free Generation of Photosensitizer Radical-Ion Pairs Enables Multipath Photoredox for Hypoxia-Tolerant Photodynamic Therapy

Weiyun Yao,[1] Linfang Yang,[1] Ruizhe Chen,[1] Siwei Yao,[1] Haolin Zhang,[1] Mingxuan Jia,[1] Yonghui Pan,[2] Ruida Bai,[1] Zhongzheng Yu,[3] Quli Fan,[2] Wei Huang,*[1,2] and Wenbo Hu*[1]*

These authors contributed equally: Weiyun Yao, Linfang Yang, Ruizhe Chen, Siwei Yao.

Affiliations

[1] State Key Laboratory of Flexible Electronics (LoFE), Frontiers Science Center for Flexible Electronics, Institute of Flexible Electronics (IFE), Northwestern Polytechnical University, Xi'an 710072, China.

[2] State Key Laboratory of Flexible Electronics (LoFE) & Institute of Advanced Materials (IAM), Nanjing University of Posts & Telecommunications, Nanjing 210023, China.

[3] Cavendish Laboratory, University of Cambridge, Cambridge CB3 0HE, United Kingdom.

Corresponding author

Correspondence: iamqlfan@njupt.edu.cn; vc@nwpu.edu.cn; iamwbhu@nwpu.edu.cn

Supporting Information Text

Chemicals and Reagents:

Aminophenyl fluorescein (APF) were purchased from Sigma-Aldrich. DMSO, DMF, chloroform and CCK-8 were obtained from AbMole Bioscience. 1,6-Dibromohexane, 3,6-di(thiophen-2-yl)-2,5-dihydropyrrolo[3,4-c]pyrrole-1,4-dione, 3,6-Bis(5-bromo-2-thienyl)-2,5-dihexyl-2,5-dihydropyrrolo[3,4-*c*]pyrrole-1,4-dione, diethylamine, bromoethane, pyridine, potassium hydroxide, vitamin E, ABDA, boron tribromide, THF, heavy-oxygen water ($H_2{}^{18}O$), 2,4-dinitrochlorobenzene (DNCB), N-hexylpyridinium, and tetrabutylammonium hexafluorophosphate ([Bu_4N] [PF_6]) were supplied by Adamas-beta®. The Agarose (A8201), was purchased from Beijing Solarbio Science & Technology Co., Ltd. Tryptone and yeast extract were purchased from OXOID. Dulbecco's Modified Eagle Medium (DMEM) solution and Phosphate Buffered Saline (PBS) were provided by Cytiva. FBS was purchased from Animal Blood Ware. Trypsin solution (0.25%) was obtained from Beijing Labgic Technology Corp., Ltd. The live/dead staining kit (SYTO9/PI) was purchased from Shanghai Maokang Biotechnology Co., Ltd. The 2',7'-Dichlorodihydrofluorescein diacetate (DCFH-DA), and dihydroethidium (DHE) were purchased from Jiangsu Kaiji Biotechnology Co., Ltd. All chemicals were used without further purification unless otherwise stated.

Instruments:

^{1}H-NMR, ^{13}C-NMR and NOESY were analyzed by Nuclear Magnetic Resonance spectrometer (ADVANCE NEO 500, Bruker, Germany). Electrochemical experiments were performed on a Multi Autolab M204 workstation (Metrohm AUTOLAB, Netherlands). Dissolved oxygen was measured using a portable oxygen meter (JPBJ-608, INESA Scientific Instrument, China). High-resolution mass spectra were acquired on a Xevo G2-XS TOF mass spectrometer (Waters, USA). Confocal fluorescence images were collected on a C2+ laser-scanning confocal microscope (Nikon, Japan). Cell viability was measured using a Spark multimode microplate reader (Tecan, Switzerland).

Material synthesis and characterization

Synthesis of DBr

Under an argon atmosphere, 1,6-dibromohexane (3.4 mL, 27 mmol), 3,6-di(thiophen-2-yl)-2,5-dihydropyrrolo[3,4-c] pyrrole-1,4-dione (1.0 g, 3.3 mmol), and potassium hydroxide (495 mg, 8.81 mmol) were dissolved in N, N-dimethylformamide (DMF, 27 mL). The reaction mixture was stirred at 65 °C overnight. After cooling to room temperature, the mixture was poured into water (200 mL), and the product was extracted with dichloromethane. The combined organic layers were washed with saturated aqueous sodium chloride, dried over anhydrous sodium sulfate, and concentrated under reduced pressure. The crude product was purified by column chromatography on silica gel using petroleum ether/dichloromethane (2:1) as the eluent to afford DBr as a black solid (600 mg, 30% yield). ^{1}H NMR (500 MHz, $CDCl_3$) δ 8.92 (dd, J = 3.9, 1.2 Hz, 2H), 7.65 (dd, J = 5.0, 1.2 Hz, 2H), 7.30 (dd, J = 5.0, 3.9 Hz, 2H), 4.14-4.05 (m, 4H), 3.40 (t, J = 6.8 Hz, 4H), 1.92-1.83 (m, 4H), 1.77 (p, J = 7.6 Hz, 4H), 1.54-1.46 (m, 8H). ^{13}C NMR (126 MHz, $CDCl_3$) δ 160.35, 138.98, 134.33, 129.74, 128.64, 127.68, 106.67, 40.96, 32.69, 31.57, 28.74, 26.76, 25.01.

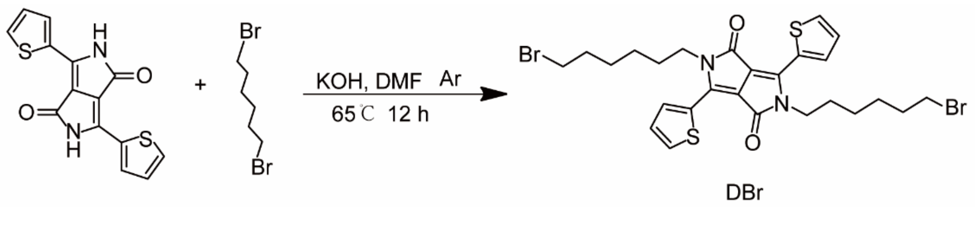

Scheme S1. Synthesis of DBr.

Synthesis of BDBr

DBr (500 mg, 0.8 mmol) was dissolved in chloroform (13 mL), and N-bromosuccinimide (NBS, 284.7 mg, 1.6 mmol) was added rapidly. The reaction mixture was stirred at room temperature in the dark for 6 h. Upon completion, the

mixture was extracted with chloroform. The organic layer was washed with saturated aqueous sodium chloride, dried over anhydrous sodium sulfate, and concentrated under reduced pressure. BDBr was obtained as a solid (400 mg, 60% yield) and used directly in the next step without further purification. ^{1}H NMR (500 MHz, $CDCl_3$) δ 8.68 (d, J = 4.2 Hz, 2H), 7.25 (d, J = 4.2 Hz, 2H), 4.03-3.98 (m, 4H), 3.40 (t, J = 6.8 Hz, 4H), 1.90-1.84 (m, 4H), 1.74 (p, J = 7.7 Hz, 4H), 1.52-1.40 (m, 8H). ^{13}C NMR (126 MHz, $CDCl_3$) δ 160.02, 137.94, 134.44, 130.72, 129.95, 118.25, 106.78, 41.02, 32.63, 31.53, 28.81, 26.75, 24.99.

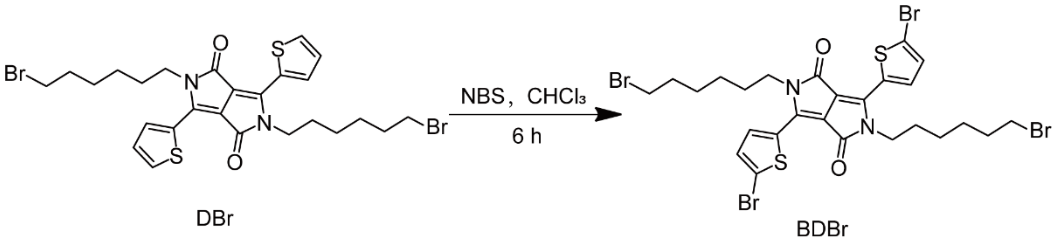

Scheme S2. Synthesis of BDBr.

Synthesis of D-Py

DBr (100 mg, 0.16 mmol) was dissolved in anhydrous THF (15 mL) in a round-bottom flask, and pyridine (10 mL, 12.4 mmol) was then added under stirring. The flask was sealed and heated under reflux at 65 °C for 24 h. After completion of the reaction, the crude product was collected by filtration and washed three times with THF. D-Py was obtained as a black-red solid (80 mg, 80% yield). ^{1}H NMR (500 MHz, DMSO-d_6) δ 9.11-9.05 (m, 4H), 8.80 (dd, J = 3.9, 1.2 Hz, 2H), 8.60 (td, J = 7.9, 1.4 Hz, 2H), 8.19-8.10 (m, 6H), 7.42 (dd, J = 5.0, 3.9 Hz, 2H), 4.59 (td, J = 7.6, 2.8 Hz, 4H), 4.00 (t, J = 7.6 Hz, 4H), 1.91 (p, J = 7.5 Hz, 4H), 1.63 (p, J = 7.8 Hz, 4H), 1.41-1.28 (m, 8H). ^{13}C NMR (126 MHz, DMSO-d_6) δ 160.34, 145.48, 144.71, 139.27, 134.67, 133.02, 129.02, 128.74, 128.07, 106.51, 60.69, 41.23, 30.52, 29.14, 25.63, 25.03. $[M]^{2+}$: calculated for 312.13, found 312.31.

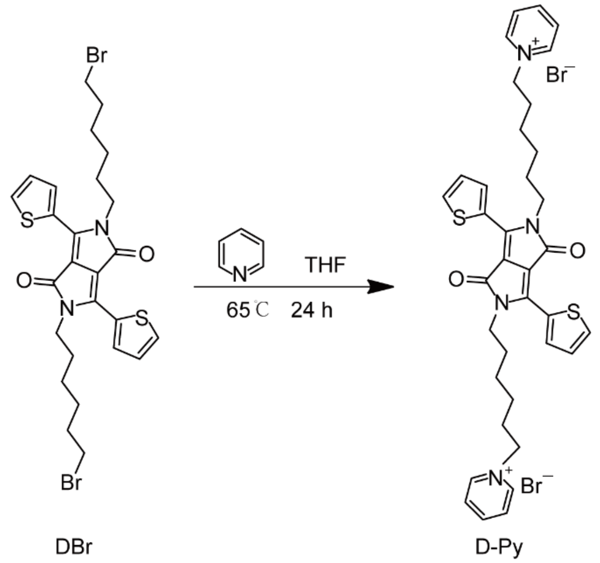

Scheme S3. Synthesis of D-Py.

Synthesis of D-TE

BDBr (300 mg, 0.36 mmol) was dissolved in THF (80 mL) in a round-bottom flask, and diethylamine (20 mL, 0.194 mol) was then added. The reaction mixture was heated under reflux for 12 h. After completion, the mixture was extracted three times with dichloromethane and water. The combined organic layers were dried over anhydrous

sodium sulfate, filtered, and concentrated under reduced pressure. The crude product was purified by column chromatography using dichloromethane/methanol (100:1) as the eluent to afford D-TE as a purple solid (100 mg, 36% yield). ^{1}H NMR (500 MHz, $CDCl_3$) δ 8.67 (d, J = 4.2 Hz, 2H), 7.24 (d, J = 4.2 Hz, 2H), 3.99 (t, J = 7.7 Hz, 4H), 2.58 (q, J = 7.3 Hz, 8H), 2.45 (d, J = 7.9 Hz, 4H), 1.71 (q, J = 7.7 Hz, 4H), 1.52-1.33 (m, 12H), 1.05 (t, J = 7.2 Hz, 12H). ^{13}C NMR (126 MHz, $CDCl_3$) δ 161.06, 138.99, 135.41, 131.70, 131.08, 119.22, 107.83, 52.60, 46.85, 42.17, 29.99, 27.23, 26.76, 26.44, 11.32. $[M+H]^+$: calculated for 768.16, found 769.30.

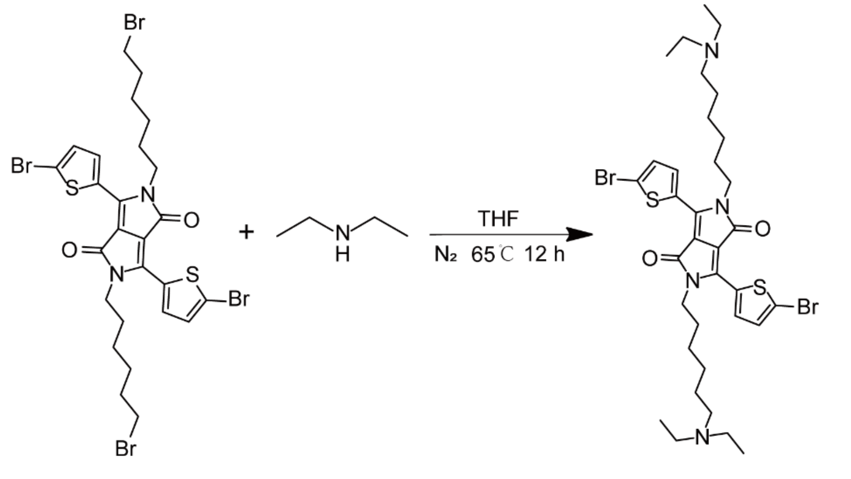

Scheme S4. Synthesis of D-TE.

Synthesis of DB-TE

D-TE (100 mg, 0.13 mmol) and bromoethane (10 mL, 0.13 mol) were dissolved in toluene. The reaction mixture was stirred under reflux for 12 h. After completion, the solvent was removed under reduced pressure. The crude product was purified by column chromatography using dichloromethane/methanol (50:1) as the eluent to afford DB-Py as a purple solid (53 mg, 49% yield). ^{1}H NMR (500 MHz, DMSO-d_6) δ 8.56 (d, J = 4.3 Hz, 2H), 7.60 (d, J = 4.3 Hz, 2H), 3.96 (t, J = 7.3 Hz, 4H), 3.21 (q, J = 7.2 Hz, 12H), 3.08 (t, J = 8.3 Hz, 4H), 1.60 (dt, J = 38.3, 7.6 Hz, 8H), 1.42-1.30 (m, 8H), 1.15 (t, J = 7.2 Hz, 18H). ^{13}C NMR (126 MHz, DMSO- d_6) δ 160.63, 138.65, 135.26, 132.70, 131.11, 119.78, 107.39, 56.35, 52.39, 41.76, 29.58, 26.22, 25.86, 21.32, 7.63. $[M]^{2+}$: calculated for 413.11, found 413.38.

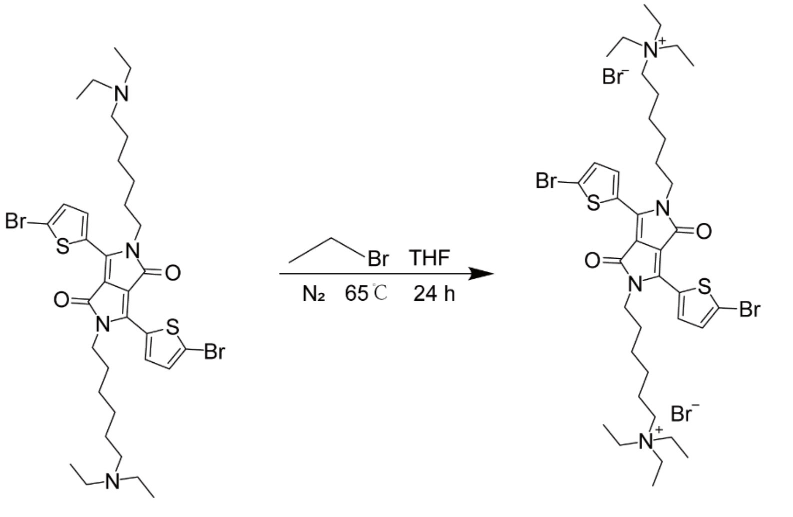

Scheme S5. Synthesis of DB-TE.

Synthesis of DB-Py

BDBr (100 mg, 0.12 mmol) was dissolved in anhydrous tetrahydrofuran (THF, 25 mL) in a round-bottom flask. Pyridine (10 mL, 124 mmol) was then added under stirring. The flask was sealed and heated under reflux at 65 °C for 24 h. After completion of the reaction, the product was collected by filtration and washed three times with THF. DB-Py was obtained as a black-red solid (75 mg, 80% yield). ^{1}H NMR (500 MHz, DMSO-d_6) δ 9.10-9.05 (m, 4H), 8.60 (tt, J = 7.8, 1.4 Hz, 2H), 8.53 (d, J = 4.2 Hz, 2H), 8.18-8.13 (m, 4H), 7.58 (d, J = 4.2 Hz, 2H), 4.59 (t, J = 7.4 Hz, 4H), 3.93 (t, J = 7.5 Hz, 4H) , 1.91 (p, J = 7.5 Hz, 4H), 1.61 (p, J = 7.6 Hz, 4H), 1.39-1.28 (m, 8H). ^{13}C NMR (126 MHz, DMSO-d_6) δ 160.58, 145.96, 145.21, 138.63, 135.25, 132.69, 131.07, 128.56, 119.74, 107.37, 61.17, 41.74, 31.00, 29.56, 26.10, 25.48. $[M]^{2+}$: calculated for 391.04, found 391.12.

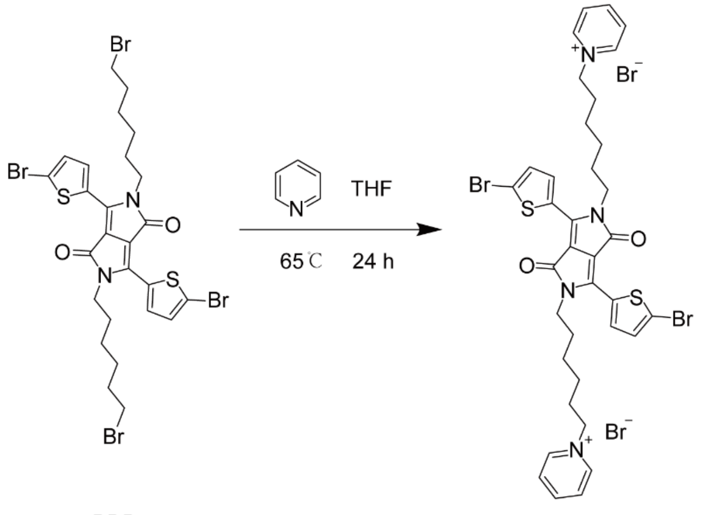

Scheme S6. Synthesis of DB-Py.

Molar extinction coefficient

A stock solution of D-Py in water with a known concentration was first prepared. The initial absorbance was adjusted to approximately 0.2 by adding an appropriate amount of the D-Py stock solution. A series of seven solutions with different concentrations was then prepared by incrementally adding known amounts of the stock solution, covering a concentration range suitable for accurate measurement. The absorbance of each solution was recorded using a UV-VIS-NIR spectrophotometer under identical conditions. Absorbance was plotted against concentration, and linear regression was used to fit the data according to the Beer–Lambert law. The slope of the fitted line was taken as the molar extinction coefficient of D-Py in water. The molar extinction coefficients of DB-TE and DB-Py were determined using the same method.

Photothermal measurements

The photothermal performance of D-Py, DB-TE, and DB-Py in H_2O (5 µg mL^{-1}, 200 µL) was evaluated under white-light irradiation at an intensity of 50 mW cm^{-2} for 5 min. After irradiation, the light was turned off, and the solution was allowed to cool naturally to room temperature. During the entire process, temperature changes were recorded every 30 s using an infrared thermal camera (Fotric 225, Shanghai, China).

Conformational searching

The representative dimer conformation of DB-Py was identified through conformational searching. First, 50 possible DB-Py dimer conformations were generated using the Genmer package. These structures were pre-optimized with the

semi-empirical MOPAC2016 program at the PM6-D3H4 level[1]. From this initial set, the 10 conformations with the lowest relative energies and distinct geometries were selected for further optimization using DFT calculations in Gaussian 16, employing the same functional and basis set as those used for ground-state geometry optimization. The optimized structures and corresponding energies of these 6 conformations are shown in Fig. S14 and Table. S1. Among them, dimer 6, which has the lowest electronic energy, was chosen as the representative dimer structure. All optimized structures were further verified by harmonic vibrational frequency calculations. Genmer, MOPAC, and Gaussian 16 were implemented through the Molclus program[2]. Wavefunction analyses, including electrostatic potential (ESP) and independent gradient model based on Hirshfeld partition (IGMH) analysis[3] were carried out using Multiwfn 3.8 (dev)[4,5]. All computational results were visualized with VMD 1.9.4 program[6].

Reactive oxygen species (ROS) measurements

ROS generation was evaluated using selective probes for different ROS species as follows.

Total ROS detection: Total ROS was quantified using 2′,7′-dichlorodihydrofluorescein diacetate (DCFH-DA). The absorbance of each sample was adjusted to a maximum of 0.2, and the final DCFH concentration was set to 40 μM. In cellular systems, DCFH-DA passively diffuses into cells and is hydrolyzed by intracellular esterases to form non-fluorescent DCFH. In cell-free systems, DCFH was generated by sodium hydroxide pretreatment. Upon irradiation with a white-light with a 495 nm long-pass filter at 50 mW cm^{-2}, DCFH was oxidized by ROS to form highly fluorescent 2′,7′-dichlorofluorescein (DCF). Fluorescence spectra were recorded every 10 s over 495–640 nm with excitation at 488 nm for a total of 60 s.

•OH detection: APF shows weak fluorescence in neutral solution but reacts with •OH to generate fluorescein, which exhibits strong fluorescence. The sample absorbance was adjusted to 0.2, and APF was used at a final concentration of 5 μM. Samples were irradiated with a white-light equipped with a 495 nm long-pass filter, with the light intensity maintained at 50 mW cm^{-2}. Fluorescence spectra were collected over 480–800 nm with excitation at 470 nm every 10 s for 60 s.

O_2^{-}• detection: O_2^{-}• was detected using DHR123, whose fluorescence intensity increases with O_2^{-}• concentration. The sample absorbance was adjusted to 0.2, and the final DHR123 concentration was 40 μM. Irradiation was provided by a white-light with a 495 nm long-pass filter at 50 mW cm^{-2}. Fluorescence spectra were recorded over 510–800 nm with excitation at 500 nm every 10 s for a total of 60 s.

$^1O_2$ detection: $^1O_2$ generation was monitored by following the absorbance decay of ABDA. An ABDA stock solution (1.5 mM) was prepared in DMSO. For each measurement, 10 μL of the ABDA stock solution was added to the photosensitizer (PS) solution in a quartz cuvette to give a final volume of 2 mL and a final ABDA concentration of 75 μM. The concentration of the PS was adjusted such that its maximum absorbance did not exceed 0.2. As a control, 10 μL of the ABDA stock solution was added to 2 mL of pure water without PS. Samples were irradiated with a white-light equipped with a 495 nm long-pass filter to remove ultraviolet and short-wavelength visible light. The irradiance at the sample position was maintained at 50 mW cm^{-2}. Absorbance spectra were recorded over 250–800 nm at 10 s intervals for a total irradiation time of 60 s.

ROS generation under hypoxic conditions: The solution containing the probe and the PS was placed in a sealed cuvette and purged with pure argon (Ar) for 20 minutes under ultrasonication to remove dissolved oxygen.

Dissolved oxygen measurement

An aqueous solution of DB-Py (10 mL, 100 μg mL^{-1}) was purged with nitrogen for 20 min under magnetic stirring to remove dissolved oxygen, and then kept in the dark for 30 minutes to reach equilibrium. The solution was subsequently irradiated with white-light at an intensity of 50 mW cm^{-2}. The dissolved oxygen concentration was monitored in real time using a portable dissolved oxygen meter (JPBJ-608, INESA Scientific Instrument, China). Measurements were recorded at defined time intervals during both dark and light periods to compare oxygen evolution in the absence and presence of irradiation.

Isotopic mass spectrometry measurement

DB-Py was separately dissolved in $H_2{}^{18}O$ and $H_2{}^{16}O$, followed by addition of an appropriate amount of ABDA. The solutions were purged with nitrogen for 20 minutes to remove dissolved oxygen and then irradiated with white-light for 30 min. Under illumination, DB-Py catalysed oxidation of $H_2{}^{18}O$ to generate $^{18}O_2$, which was subsequently sensitized to produce $^1O_2$. The generated $^1O_2$ was trapped by ABDA to form the corresponding ABDA endoperoxide (calculated molecular weight: 445.0912). After irradiation, the characteristic signals were analyzed by high-resolution mass spectrometry.

fs-TA spectra

The fs-TA spectra were recorded using a home-built transient absorption setup based on an amplified Ti:sapphire laser system (Solstice Ace, Spectra-Physics). The laser delivered 800 nm fundamental pulses with a pulse duration of 120 fs, a repetition rate of 1 kHz, and an average power of 7 W. The output beam was split into pump and probe paths in a 7:3 ratio. The pump beam, tunable from 240 to 2600 nm, was generated using an optical parametric amplifier (TOPAS, Light Conversion). For visible probe measurements, the probe beam was focused onto a 3 mm sapphire plate and filtered with an 850 nm short-pass filter to generate a white-light continuum covering 420–850 nm. For near-infrared measurements, the probe beam was focused onto a 5 mm YAG plate and filtered with an 800 nm long-pass filter to produce probe pulses spanning 800–1600 nm. The delay time between pump and probe pulses was controlled by a high-precision translation stage, providing a temporal resolution of 14 fs and an overall delay window of 8 ns. Samples were measured in a 2 mm path-length quartz cuvette. The probe beam was split into signal and reference channels before the sample to correct for probe fluctuations. Pump-induced absorption changes (ΔOD) were obtained by comparing probe spectra recorded with and without pump excitation, using a chopper to modulate the pump beam. The transient signals were detected by fiber-coupled spectrographs with linear array detectors. Data acquisition was controlled by custom software, and the kinetic traces were analyzed by multi-exponential fitting.

Bacteria culture growth

Methicillin-resistant *Staphylococcus aureus* (MRSA USA 300) was cultured in LB liquid medium at 4 °C, then centrifuged at 5000 rpm for 3 minutes to collect the bacteria. The turbidity of the bacterial suspension at 600 nm was measured using an absorption spectrophotometer, and the concentration was adjusted to 10^8 CFU mL^{-1} for subsequent experiments.

***In vitro* antibacterial performance under hypoxic conditions**

For the *in vitro* antibacterial assays, hypoxic conditions were established by placing the samples in an anaerobic gas-generating pouch system, which reduces the oxygen concentration to below 1% within a sealed chamber.

Antibacterial activity *in vitro*

MRSA at a concentration of 10^8 CFU mL^{-1} was incubated in PBS, RB, or DB-Py solution co-incubation at 37 °C for 3 hours. The mixture was then irradiated with white-light for 15 minutes and incubated for another 3 hours. Subsequently, 5 μL aliquots of ten-fold serial dilutions were spotted onto LB agar plates and incubated at 37 °C for 24 hours. After colony formation, the survival rate was calculated by dividing the number of CFUs in the treatment group by the number of CFUs in the PBS control group.

Test for ROS production in bacteria

MRSA at a concentration of 10^8 CFU mL^{-1} was incubated with DCFH-DA (10 μM) for 30 minutes. The bacterial suspension was then centered on the surface of a DB-Py solution co-incubation at 37 °C for 3 hours. Subsequent to sonication, the bacteria were collected by centrifugation and 10 μL of the bacterial suspension was placed in a confocal Petri dish and a coverslip was fixed. The fluorescence intensity within the MRSA bacteria was recorded using a laser confocal microscope (CLSM, Nikon C2+) before and after light exposure.

Bacterial live/dead staining assay

MRSA (10^8 CFU mL^{-1}) was incubated with the different treatment groups for 4 hours at 37 °C. For the light group, the suspension was irradiated with white-light (50 mW cm^{-2}) for 15 minutes after 4 hours. For the light group, the bacterial suspension was irradiated with white-light for 15 minutes after 4 hours of incubation. The dark group was incubated directly with the bacterial suspension for 4 h. Bacteria were collected by centrifugation with sonication and washed twice with 0.9% NaCl. The bacteria were then resuspended in 0.9% NaCl containing a live/dead bacterial stain and stained for 20 minutes. After staining, the suspension was centrifuged, the supernatant discarded, and 10 μL of the bacterial solution was placed at the bottom of a confocal petri dish. Coverslips were fixed and bacterial live/dead images were acquired using a confocal laser scanning microscope (CLSM, Nikon C2+).

SEM imaging

Bacterial solutions (10^8 CFU mL^{-1}) were treated with different treatments, then washed three times with PBS and fixed overnight with paraformaldehyde. After fixation, the bacteria were rinsed twice with PBS and then dehydrated with 20%, 40%, 60%, 80%, 90% and 100% ethanol solutions sequentially. The first three concentrations of ethanol were dehydrated for 10 minutes each, and the last three concentrations were dehydrated for 30 minutes each. Finally, the samples were metal-coated and analyzed using a scanning electron microscope (ZEISS Gemini SEM 300, Germany).

Modeling of infected wounds in mice

BALB/c female mice (6-8 weeks) were purchased from Shaanxi Pharmaceutical Medical Biotechnology Co., Ltd. After anesthesia, the backs of the mice were shaved, and and an 8 mm diameter wound was created using a perforator. To establish the infection model, 10 μL of MRSA bacterial solution (10^6 CFU mL^{-1}) was applied to the wound.

***In vivo* antibacterial experiments**

All mice were randomly divided into 6 groups: the PBS group, the PBS + L group, the RB group, the RB + L, the DB-Py group, and the DB-Py + L group. Twelve hours after infection, 10 μL of PBS or RB or DB-Py was sprayed directly onto the wounds. Upon drug spraying, the PBS + L, RB + L, and DB-Py + L groups were exposed to a white-light (with a 495 nm long-pass filter at 50 mW cm^{-2}) for 10 minutes. Wounds were photographed daily to monitor the progression of healing. Additionally, the infected area and body weight of each mouse were measured every day throughout the 7-day treatment period.

Cytocompatibility test

Mouse embryonic fibroblasts (NIH 3T3) were cultured in high-glucose DMEM supplemented with 10% fetal bovine serum (FBS) and 1% penicillin-streptomycin at 37 °C in a 5% CO_2 atmosphere. NIH 3T3 cells were seeded into 96-well plates at a density of 6×10^3 cells per well and incubated for 12 hours. Subsequently, the cells were treated with various concentrations of DB-Py (100 μL per well) and incubated for 24 and 48 hours. Following each incubation period, the medium was removed, and the cells were incubated with 100 μL of CCK-8 solution (diluted at a 1:9 ratio) for 30 minutes. The absorbance was then measured at 450 nm using a multimode microplate reader (TECAN Spark).

***In Vivo* biosafety assessment**

To assess the biosafety of DB-Py, all infected mice were euthanized at the end of the treatment period (day 8). Tissue organs from each group of mice were collected and stained with hematoxylin and eosin (H&E) for histological analysis of skin tissues and major organs including liver, kidney, spleen, lungs, heart and wounds. Stained tissues were imaged with a light microscope (Thermo Fisher EVOS FL Auto 2).

Statistical analysis

Statistical analysis was performed using GraphPad Prism 10.1.2 and Origin 2021 software. All data are expressed as the mean ± standard deviation (SD). Between-group analyses comparing the experiments were analyzed using two-way ANOVAs or t-test. Probability (P) values < 0.05 were considered statistically significant. (*$P < 0.05$, **$P < 0.01$, ***$P < 0.001$, ****$P < 0.0001$, and ns means no significance).

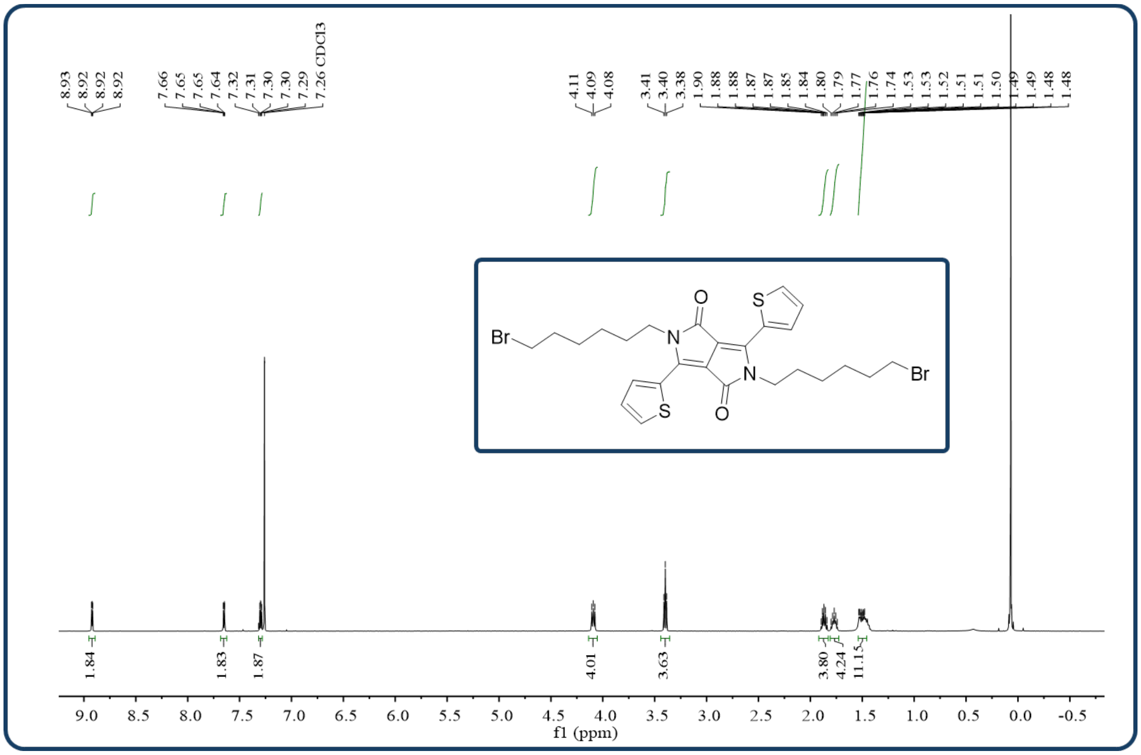

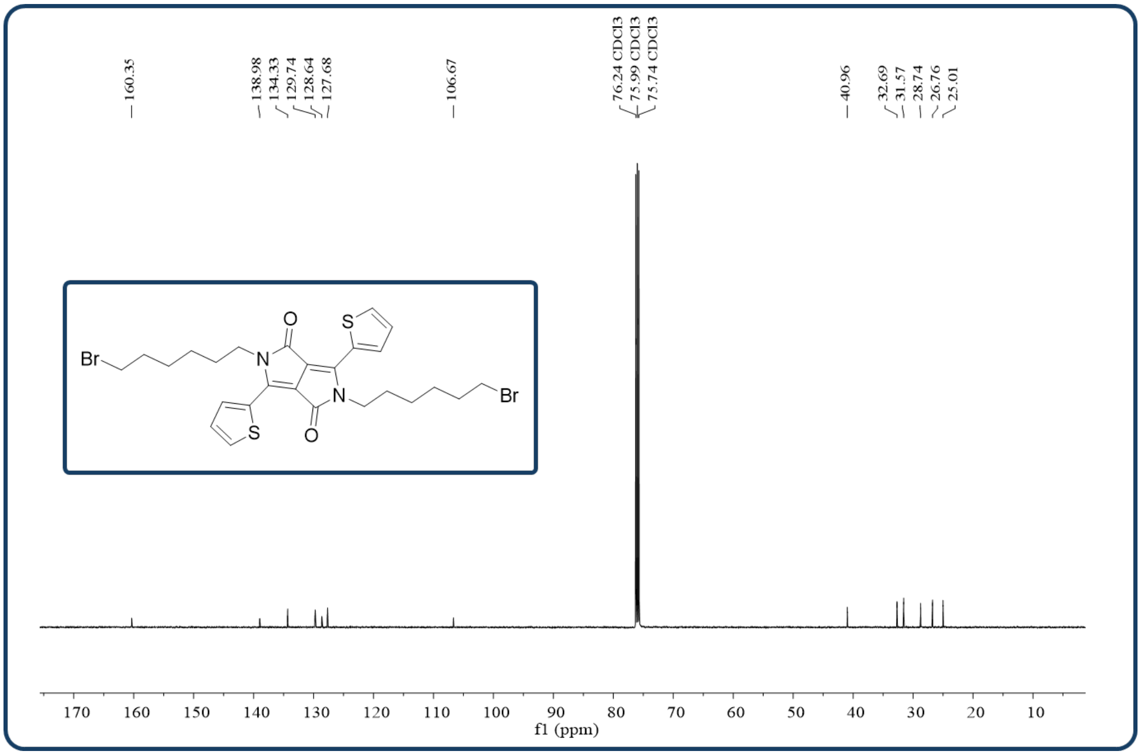

Fig. S1. ^{1}H and ^{13}C NMR spectrum of DBr.

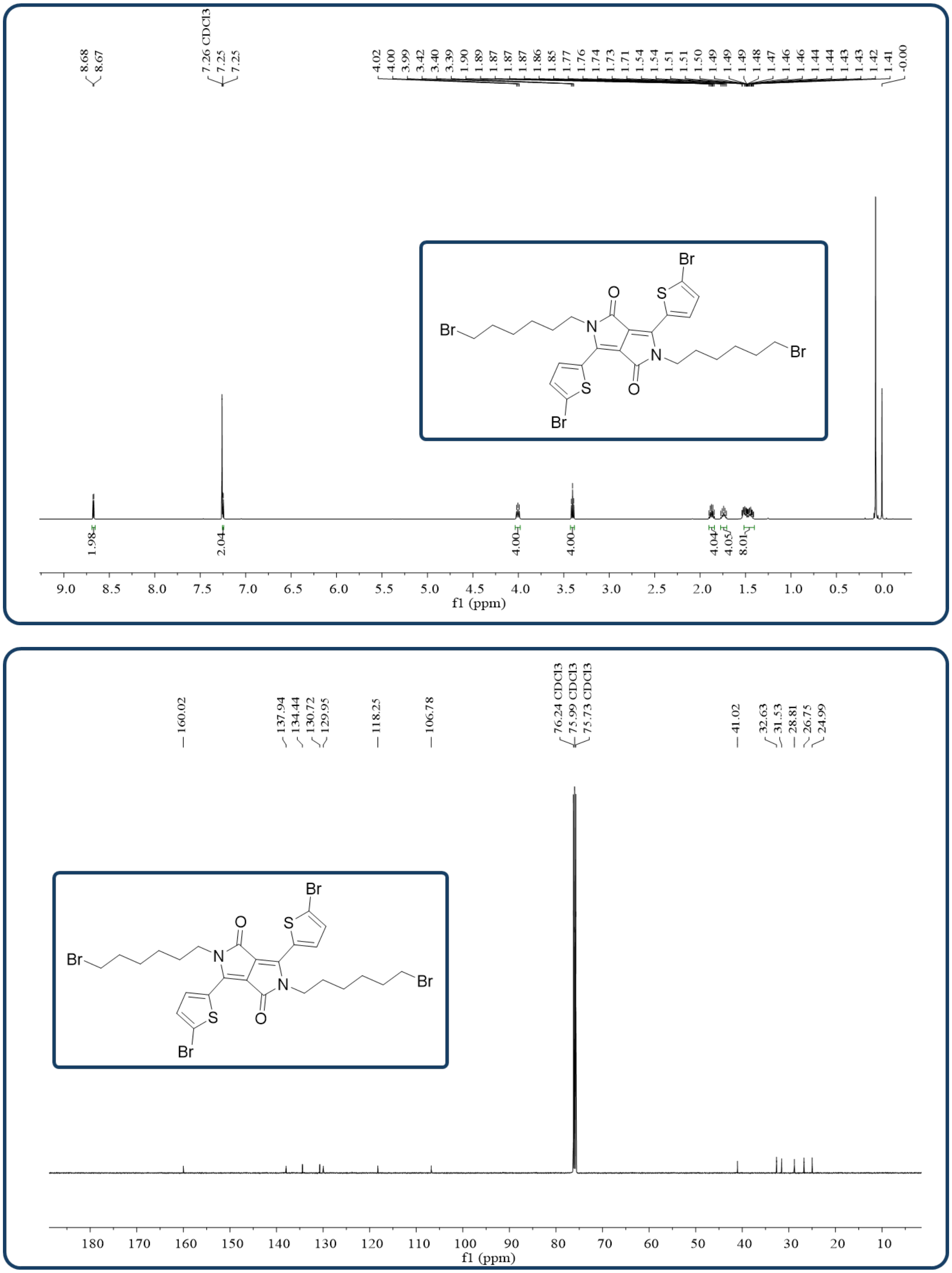

Fig. S2. ^{1}H and ^{13}C NMR spectrum of BDBr.

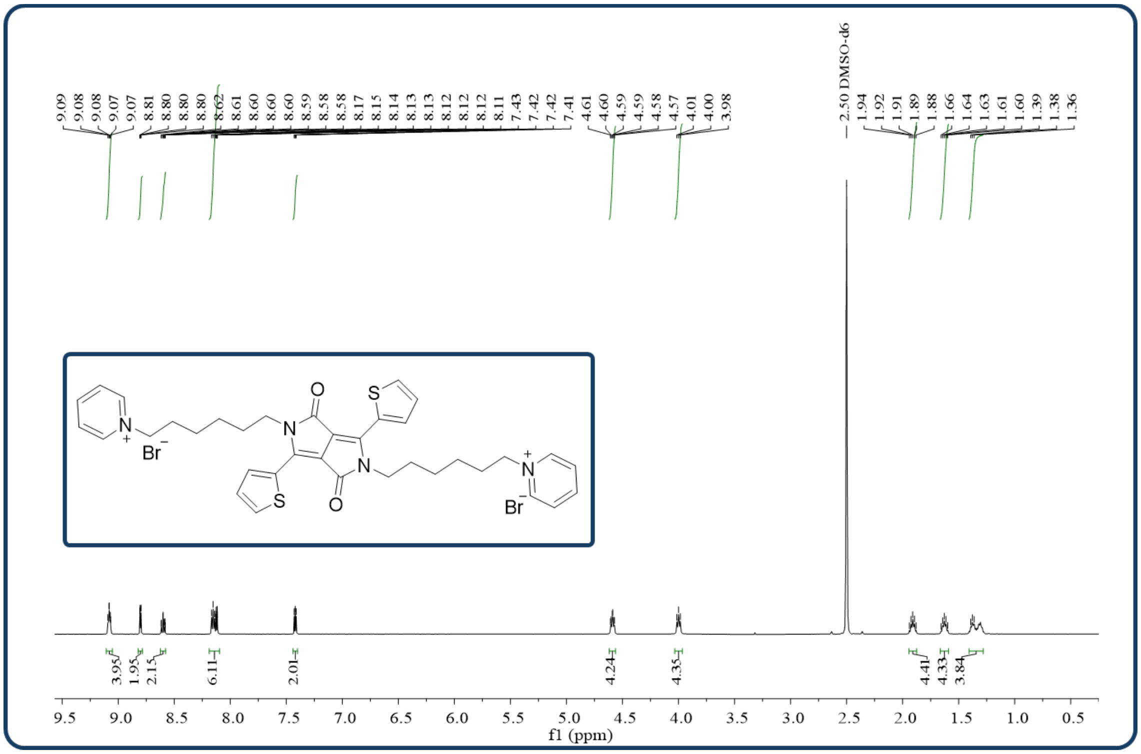

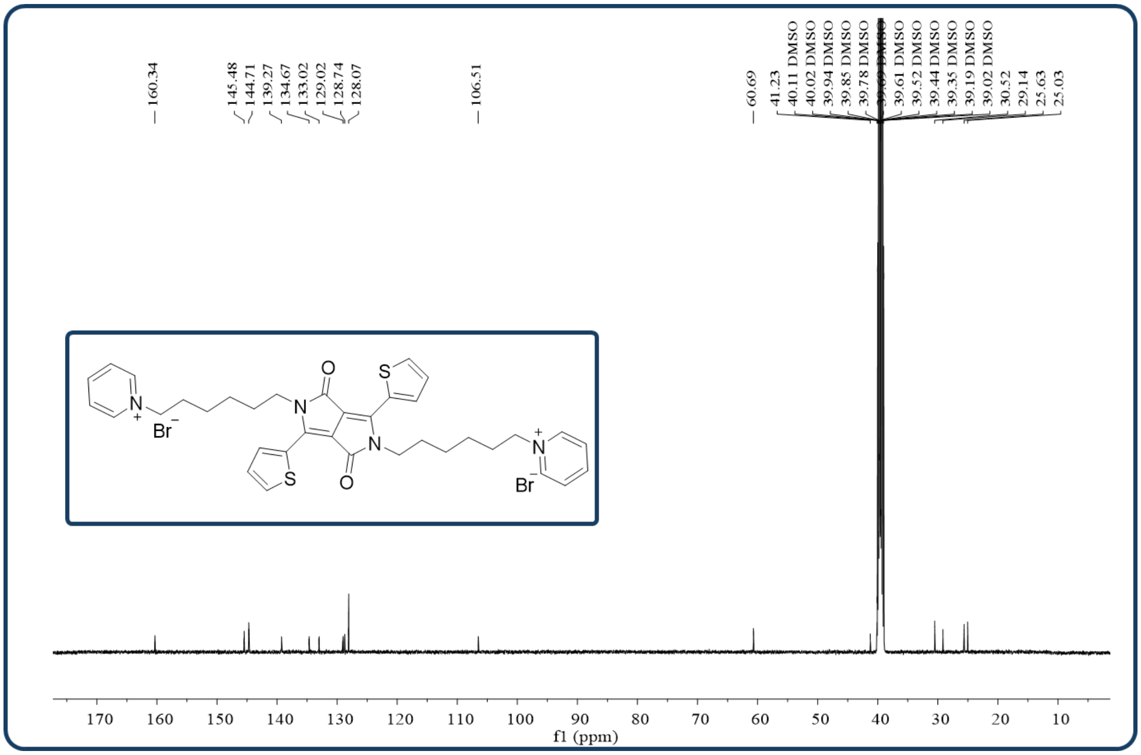

Fig. S3. ^{1}H and ^{13}C NMR spectrum of D-Py.

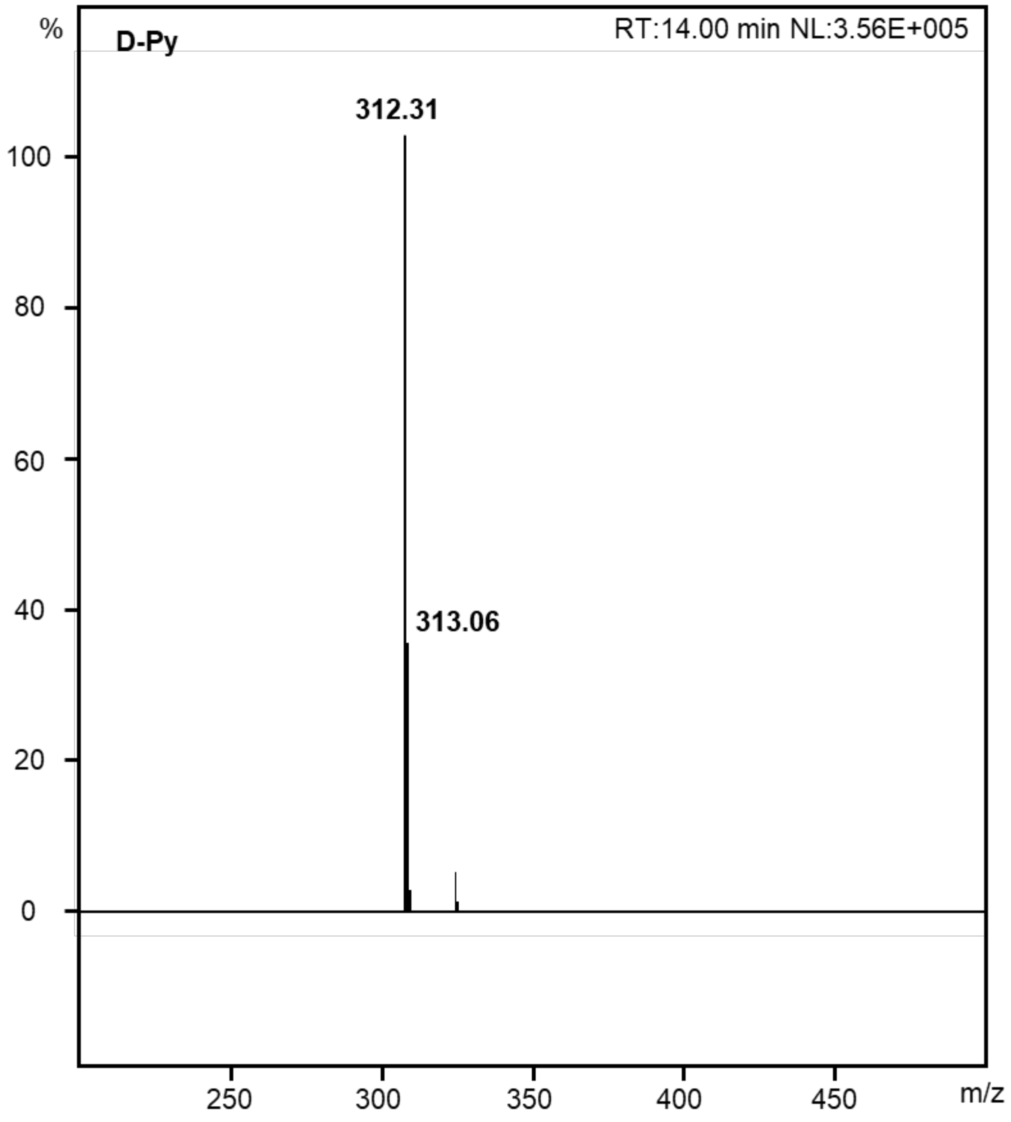

Fig. S4. Mass spectrum of D-Py.

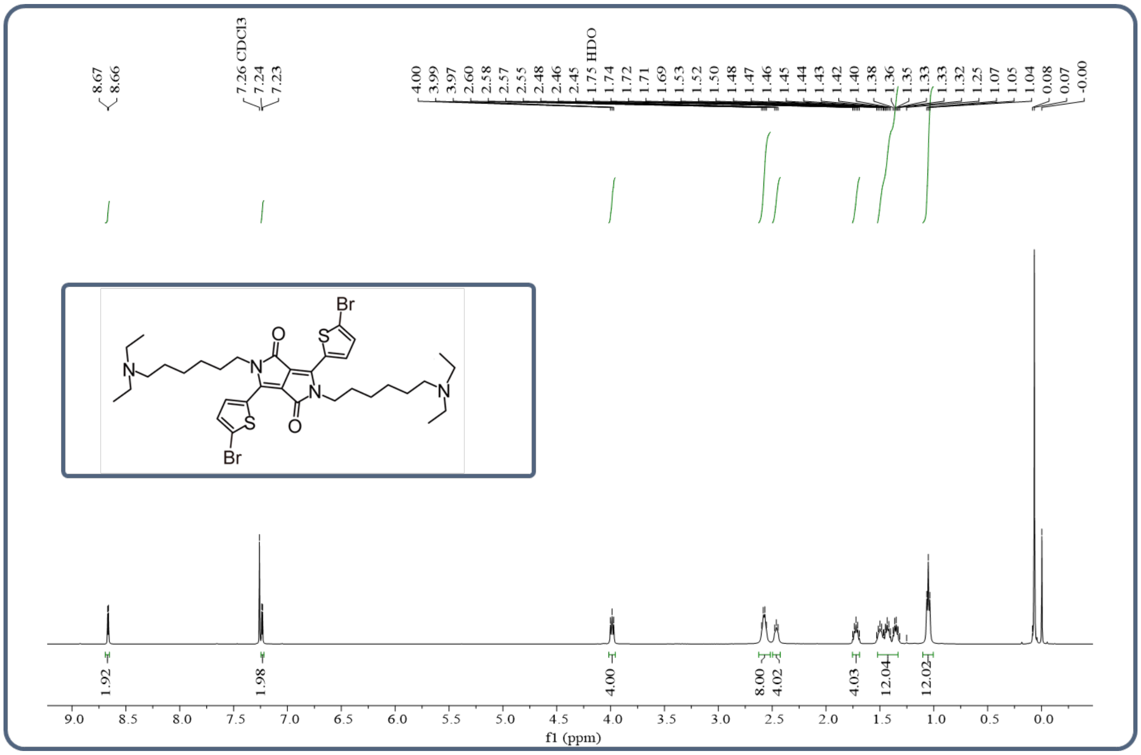

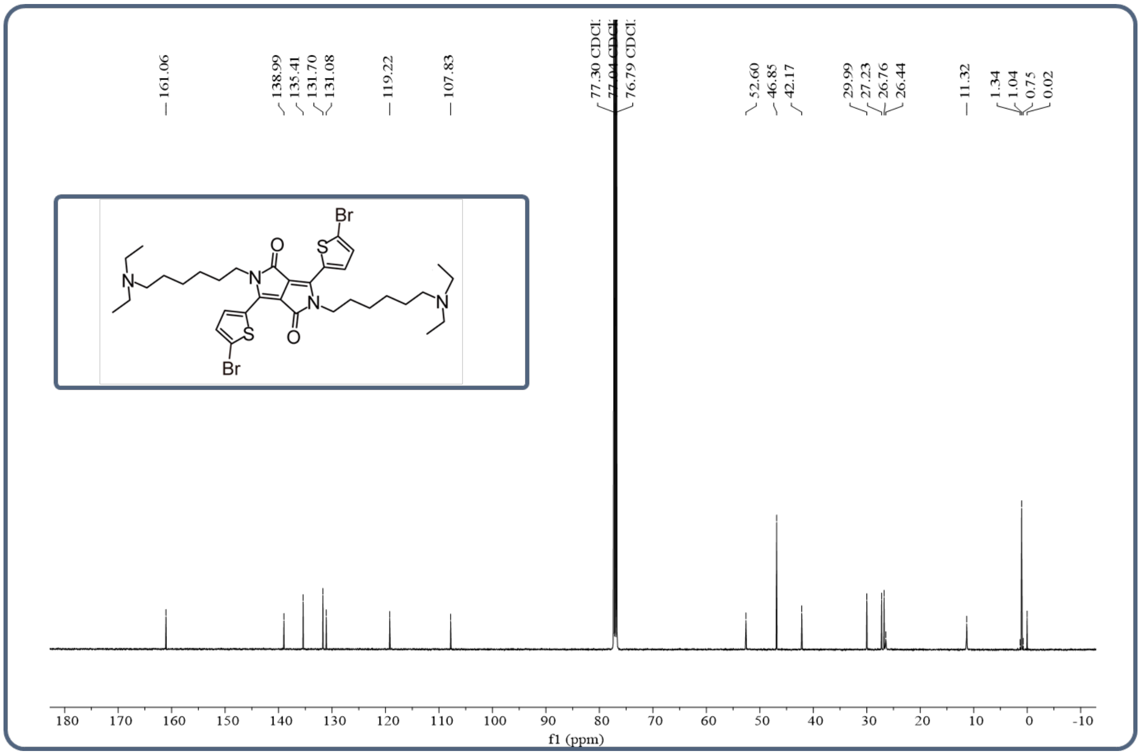

Fig. S5. ^{1}H and ^{13}C NMR spectrum of D-TE.

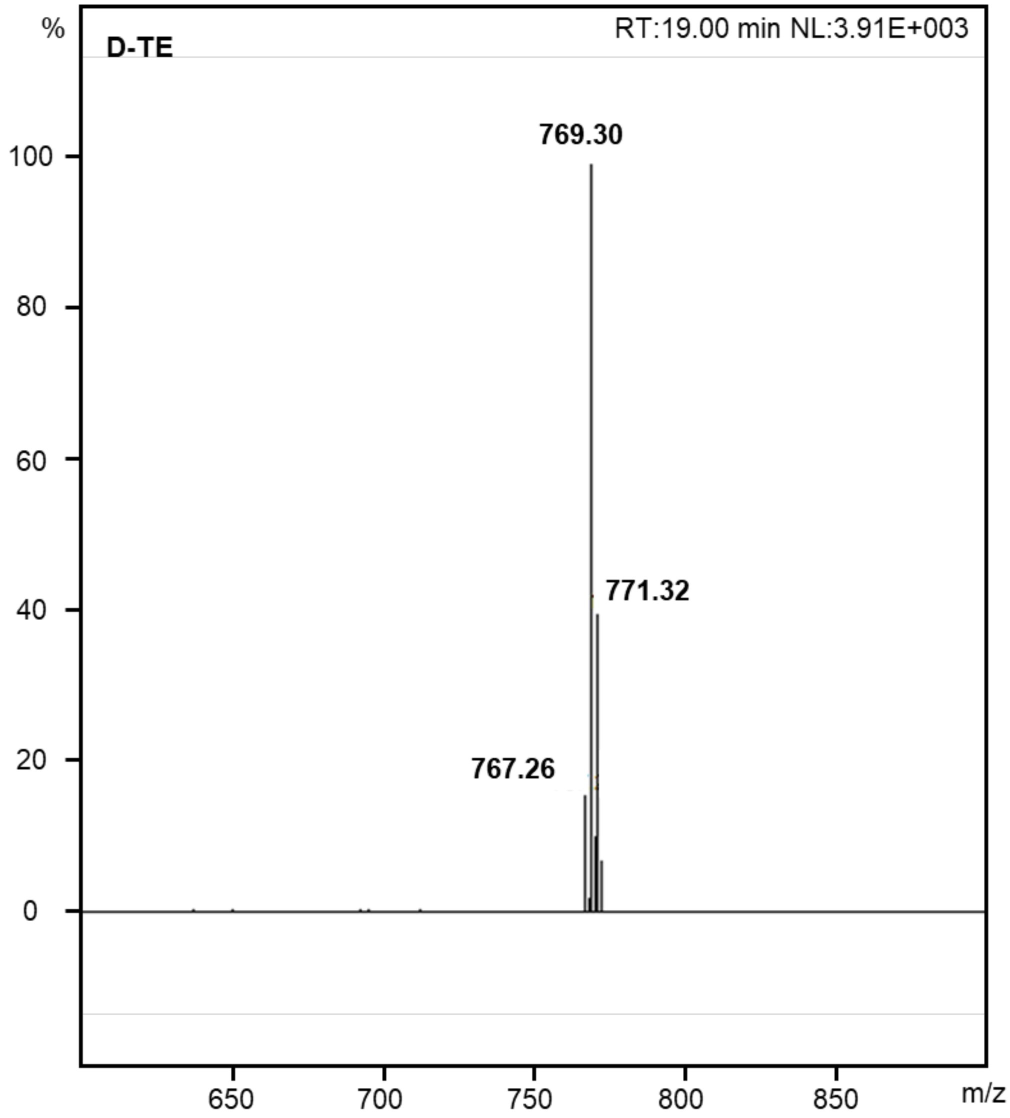

Fig. S6. Mass spectrum of D-TE.

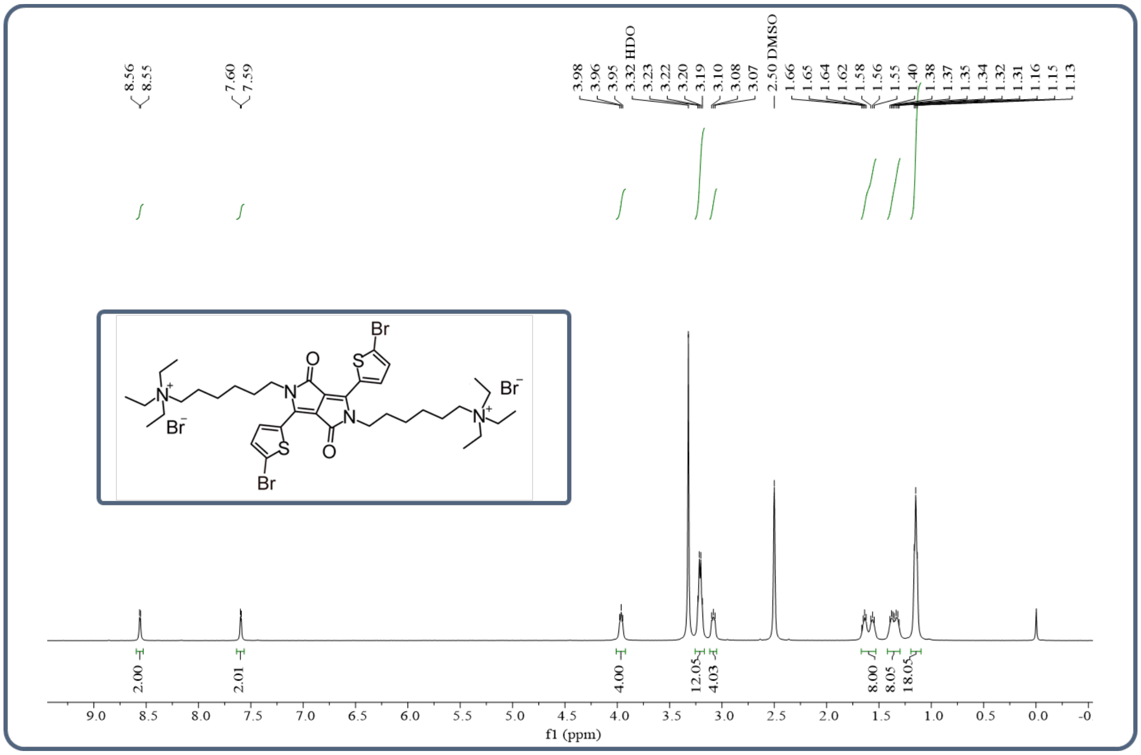

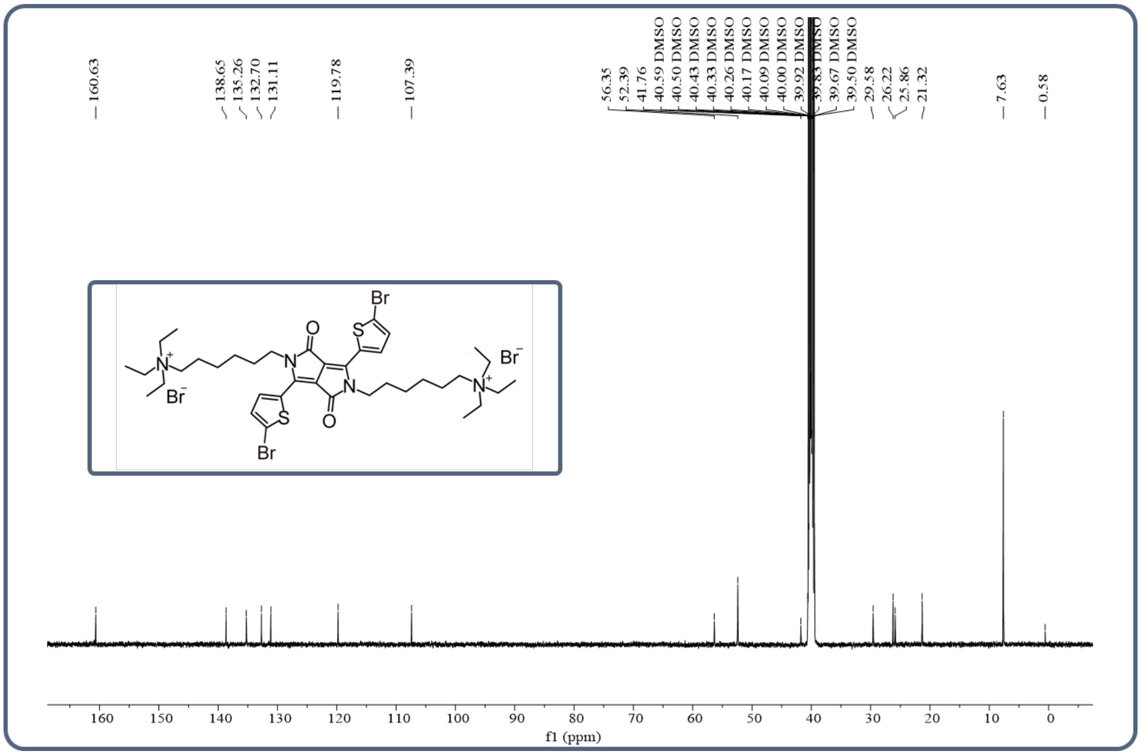

Fig. S7. ^{1}H and ^{13}C NMR spectrum of DB-TE.

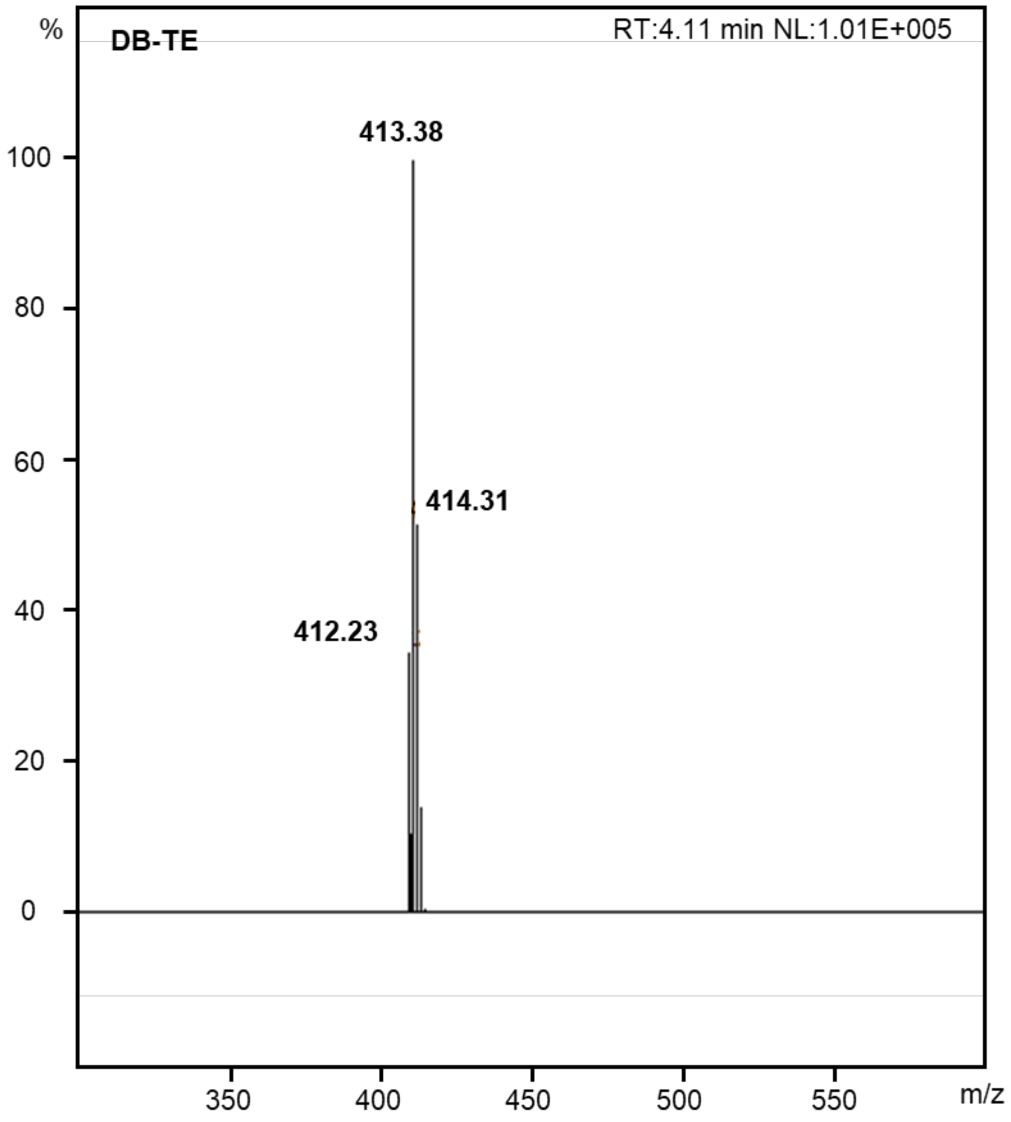

Fig. S8. Mass spectrum of DB-TE.

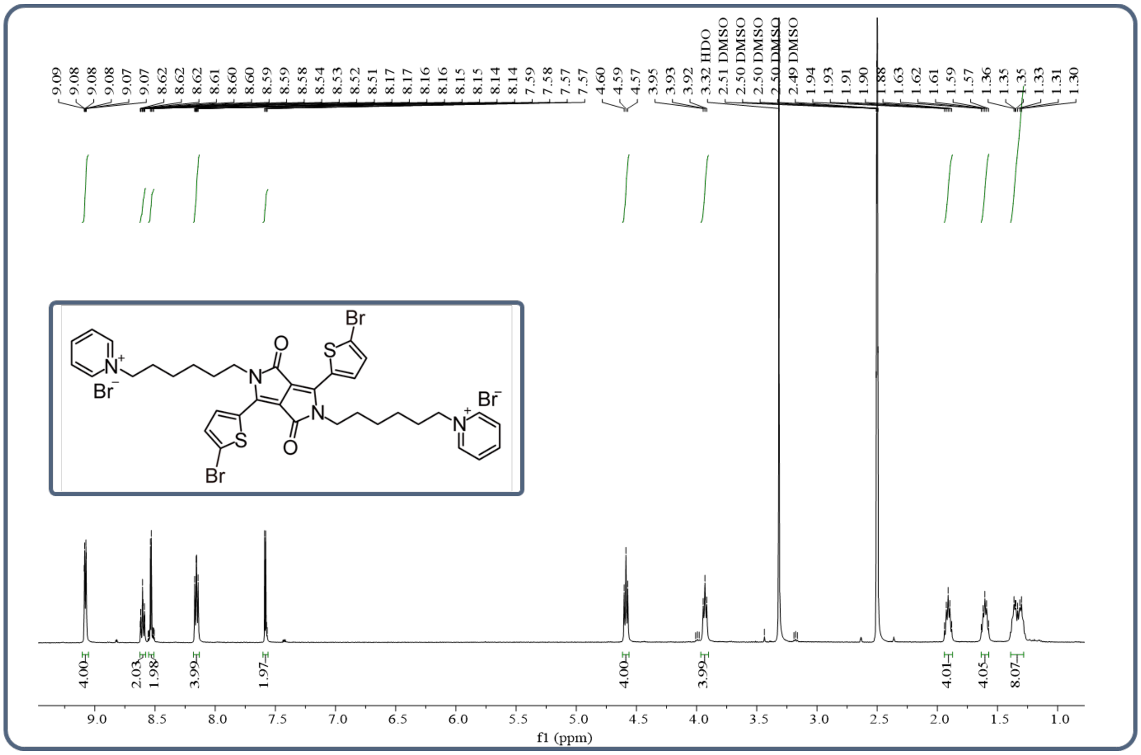

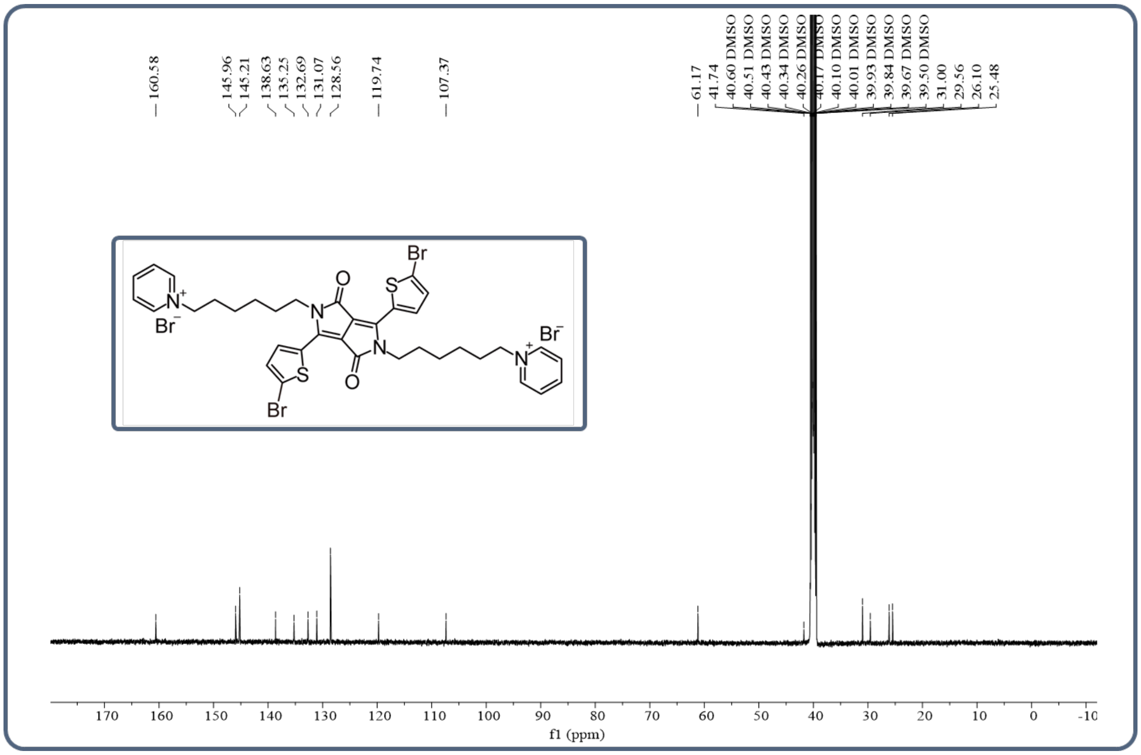

Fig. S9. ^{1}H and ^{13}C NMR spectrum of DB-Py.

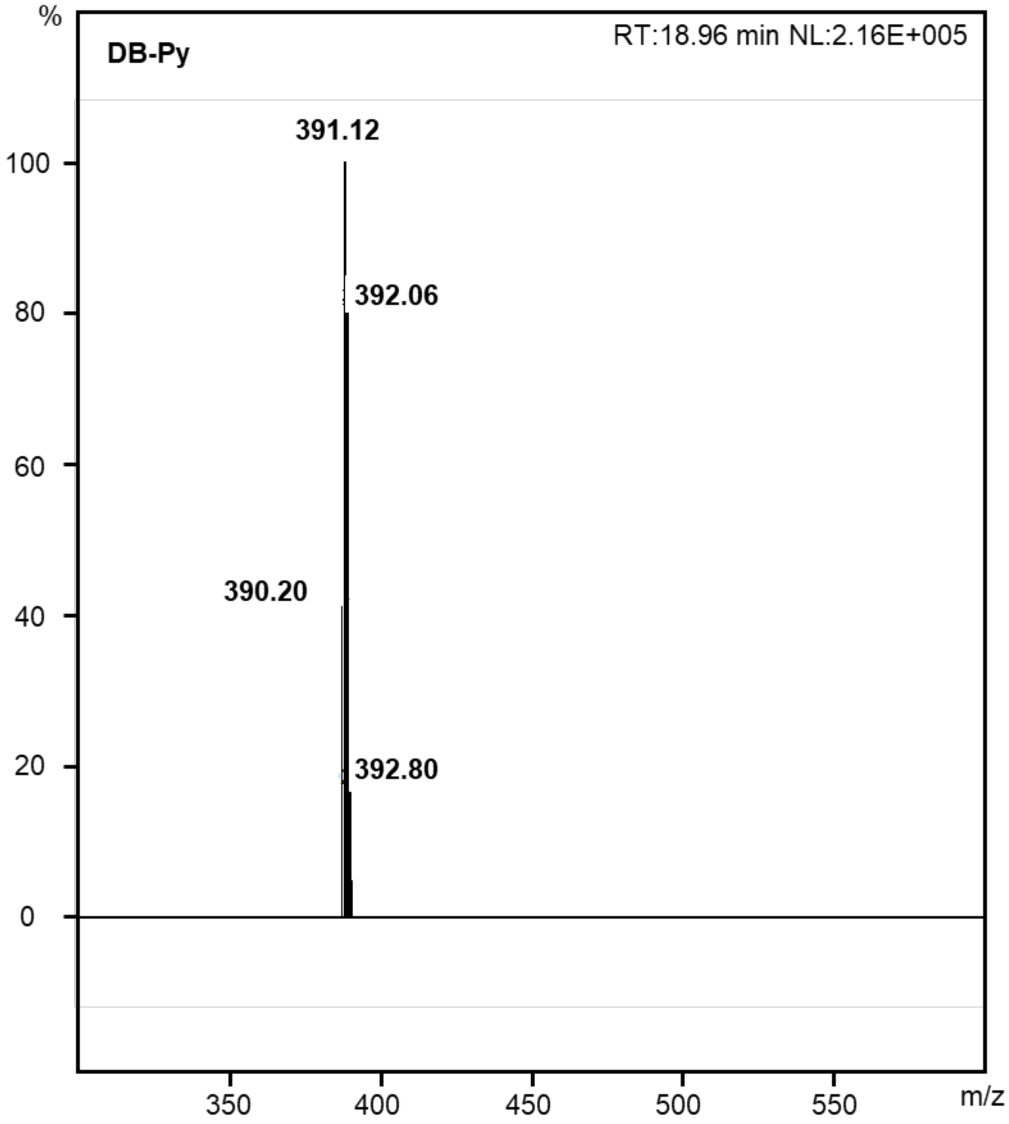

Fig. S10. Mass spectrum of DB-Py.

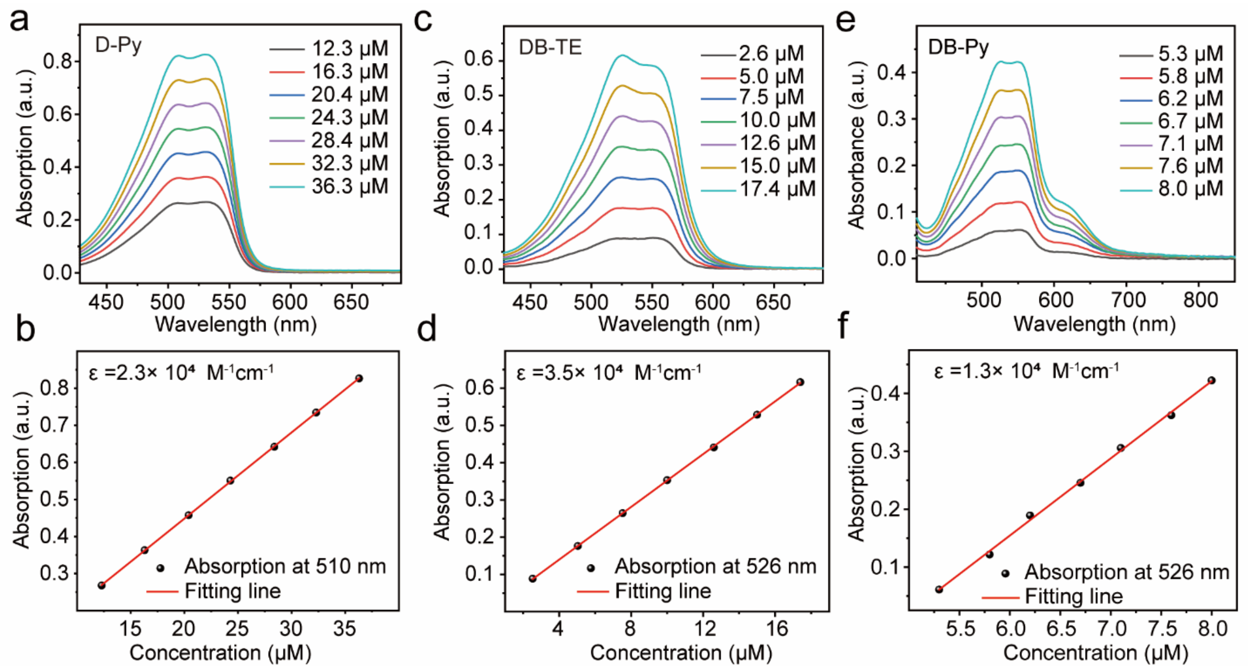

Fig. S11. Molar extinction coefficient**.** Absorption of D-Py (a), DB-TE (c) and DB-Py (e) in H_2O (with different concentration). Absorbance as a function of concentration of D-Py (b), DB-TE (d) and DB-Py (f). The molar extinction coefficients were determined to be 2.3 $\times 10^4$ M^{-1} cm^{-1}, 3.5 $\times 10^4$ M^{-1} $cm^{-1}$and 1.3$\times 10^4$ M^{-1} cm^{-1} for D-Py, DB-TE, and DB-Py, respectively.

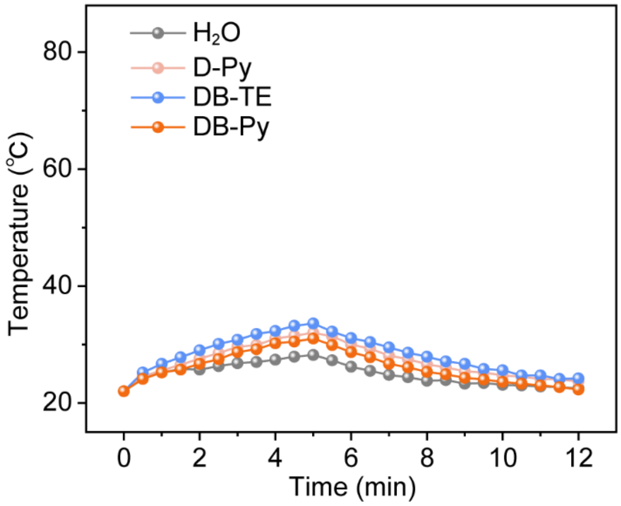

Fig. S12. Photothermic heating curves of D-Py, DB-TE and DB-Py in H_2O under white light irradiation time (50 mW cm^{-2}) for 5 min followed by the natural cooling to room temperature.

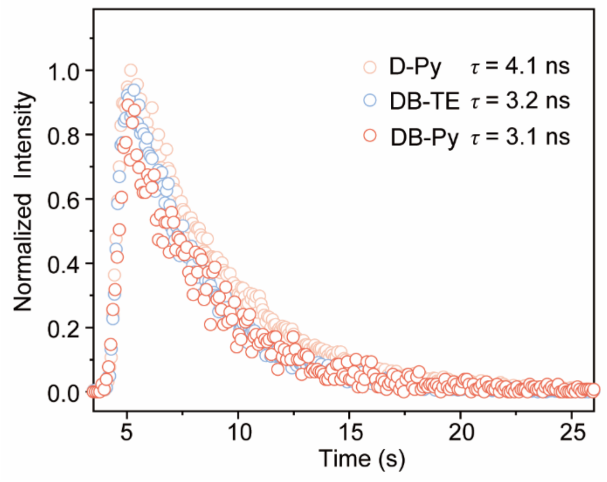

Fig. S13. PL lifetimes of D-Py at 560 nm, and DB-TE and DB-Py at 575 nm in water. (λ_{ex}: 532 nm, 80.3 ps, 1 kHz)

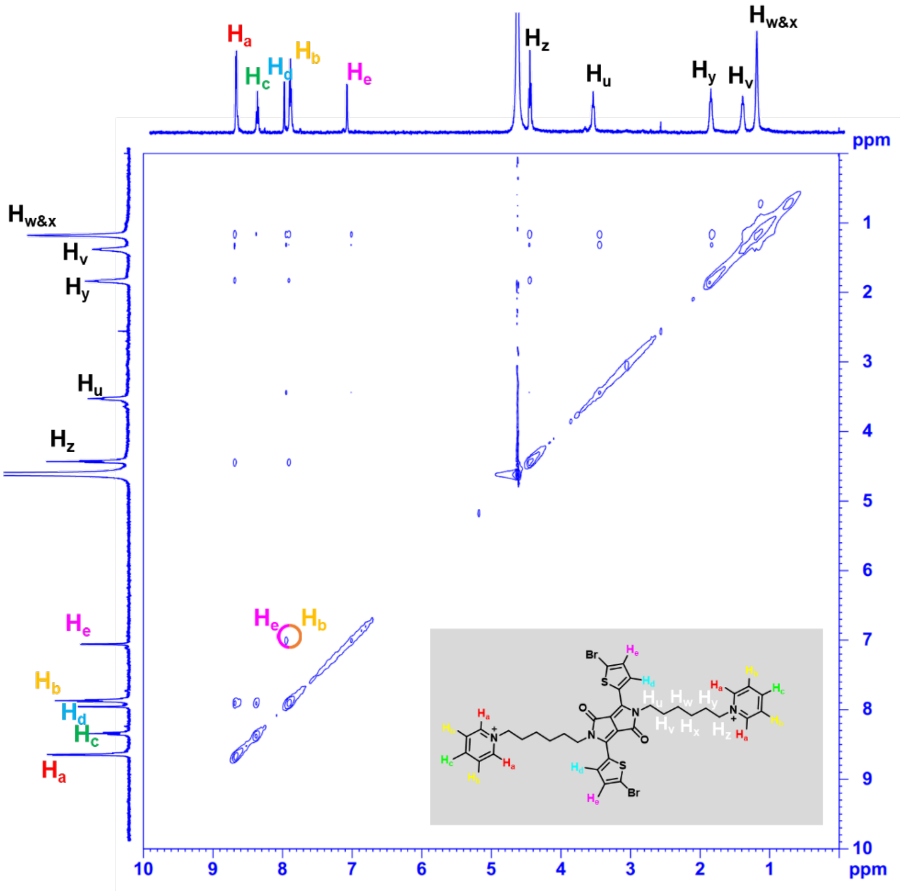

Fig. S14. The original H-H NOESY spectra of DB-Py in D_2O, presenting distinct cross-peaks between the pyridinic proton on the side chain and the protons on the DPP conjugated backbone of neighboring DB-Py molecules.

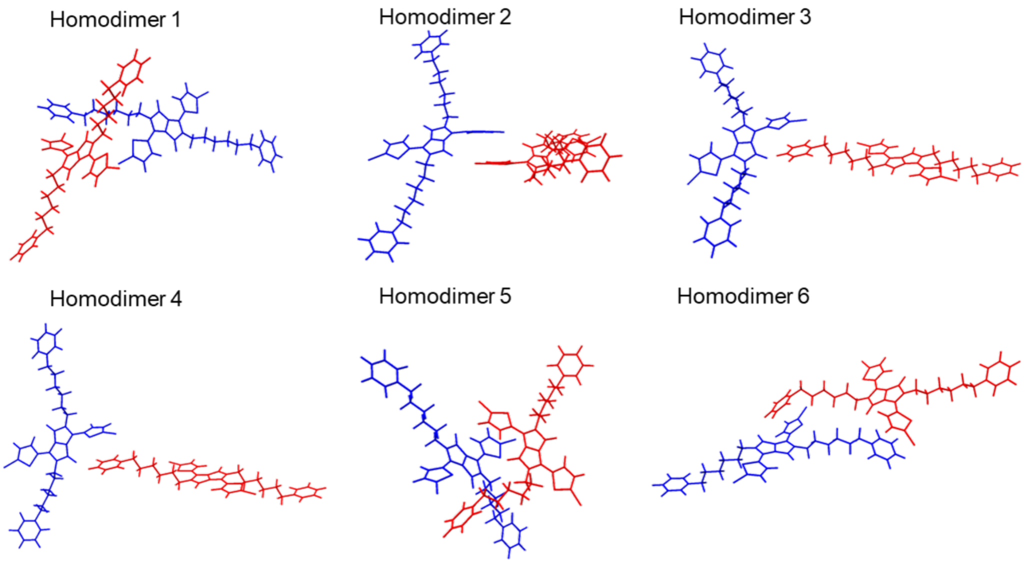

Fig. S15. Conformational searching of DB-Py homodimer. The results identify an energetically favorable homodimeric structure (homodimer 6) with an intermolecular head-to-side stacking mode, in which the side-chain pyridine lies in close spatial proximity to the DPP core of a neighboring molecule.

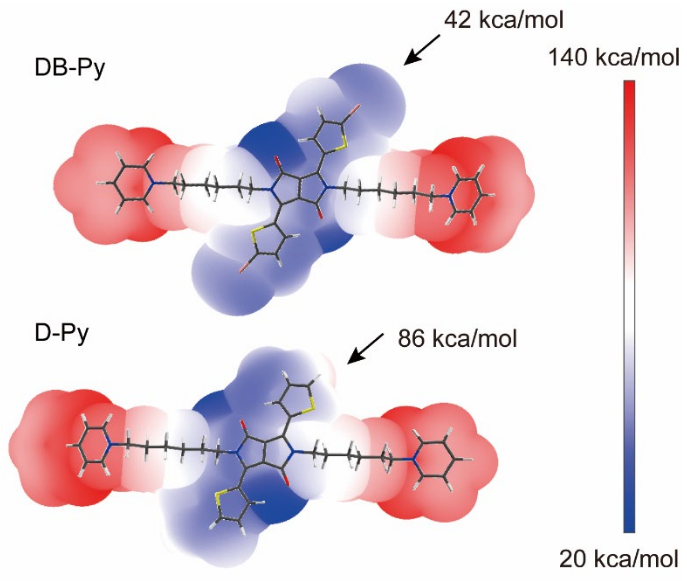

Fig. S16. Electrostatic potential (ESP) of DB-Py. The negative potential (blue region) is mainly localized at one end of the DPP backbone, while the other end exhibits a weak positive potential (red region). Bromination further lowers the potential on the thiophene fragment from 86 to 42 kcal/mol, reducing steric hindrance and strengthening electrostatic complementarity between the DPP backbone and pyridine unit, which favors head-to-side stacked homodimer in DB-Py.

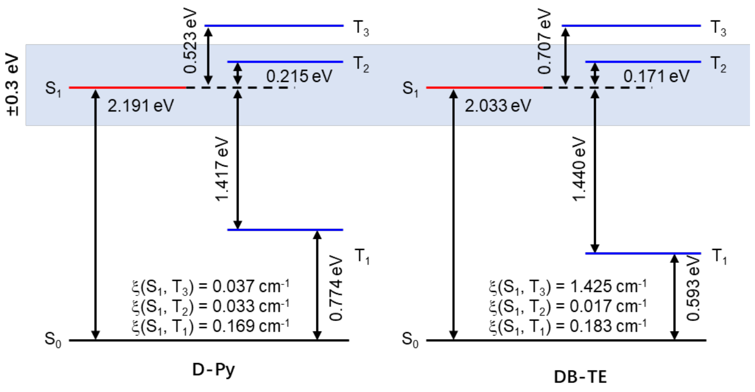

Fig. S17. Calculated intersystem crossing (ISC) channels and corresponding spin–orbit coupling (SOC) values of D-Py and DB-TE from S_1 to triplet states. The shaded regions indicate the triplet states for which the energy gap relative to S_1 (ΔE_{ST}) is less than 0.37 eV, a threshold generally considered favorable for ISC.

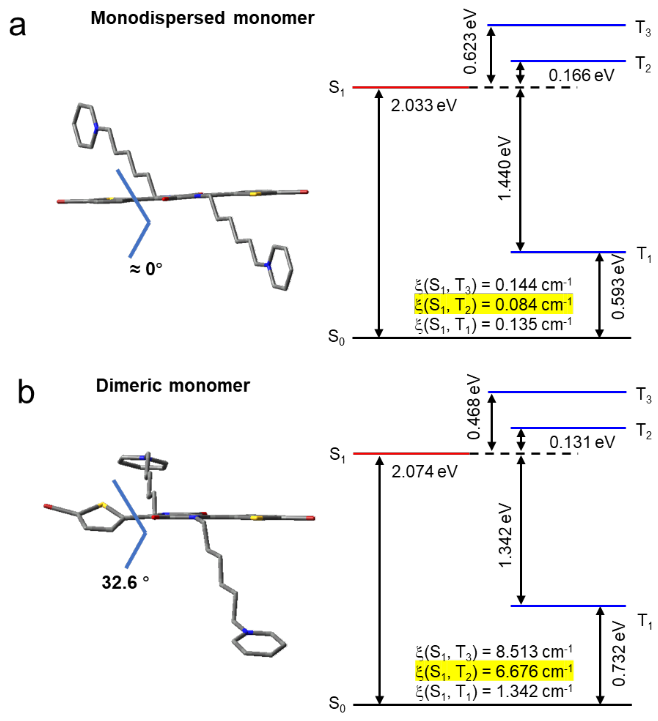

Fig. S18. Computational analysis of the ISC channels of the monodispersed DB-Py monomer (a) and the dimeric DB-Py monomer (b). Large SOC values indicate a high probability of ISC in the corresponding channels. The greater number of accessible channels together with their larger SOC values collectively suggests more facile ISC in the dimeric monomer than in the monodispersed monomer.

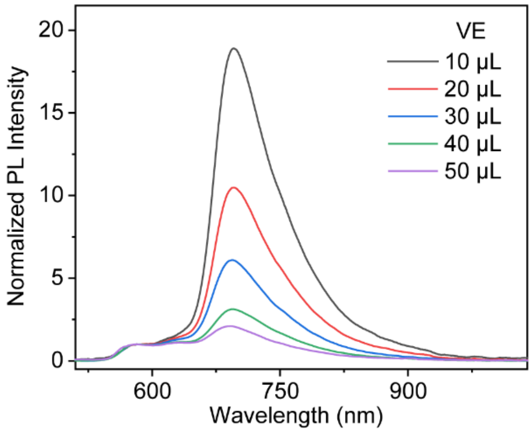

Fig. S19. DB-Py (10^{-3} M) PL spectral changes with increasing content of the triplet-state quencher VE (λ_{ex}: 532 nm). The emission intensity was normalized at 575 nm.

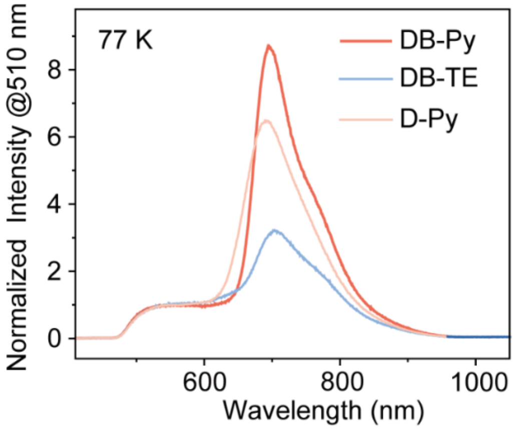

Fig. S20. PL spectra of D-Py, DB-TE and DB-Py recorded at 77K. The appearance of the 695 nm band in all three photosensitizers indicates a general tendency towards homodimer formation, with DB-Py showing the most pronounced intensity. To facilitate comparison of the relative spectral changes, the three spectra were normalized to the same intensity at 510 nm.

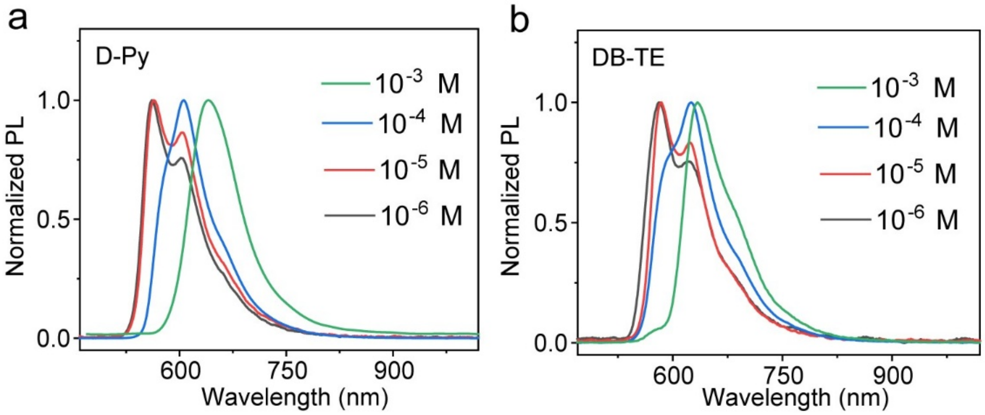

Fig. S21. Concentration-dependent PL spectra of D-Py and DB-TE in H_2O.

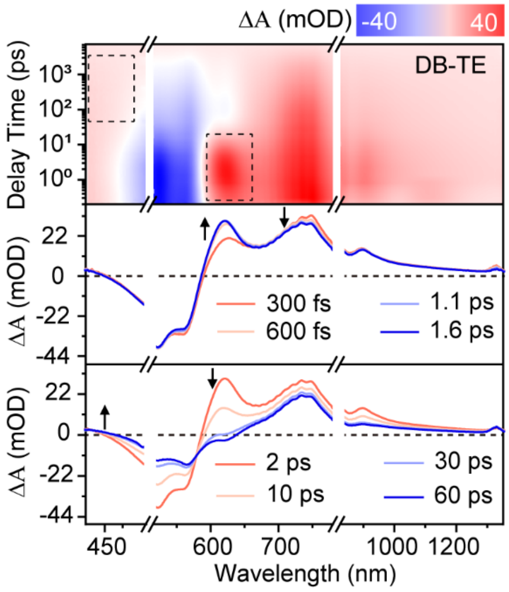

Fig. S22. fs-TA mapping of DB-TE in aqueous solution pump at 500 nm. Extracted fs-TA plots under different delay time (below). The up and down arrow indicate buildup and decay of species, respectively.

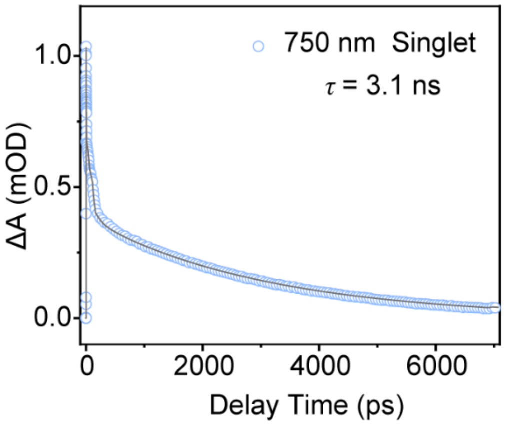

Fig. S23. Kinetic traces for DB-Py at the wavelength within ESA singlet (750 nm).

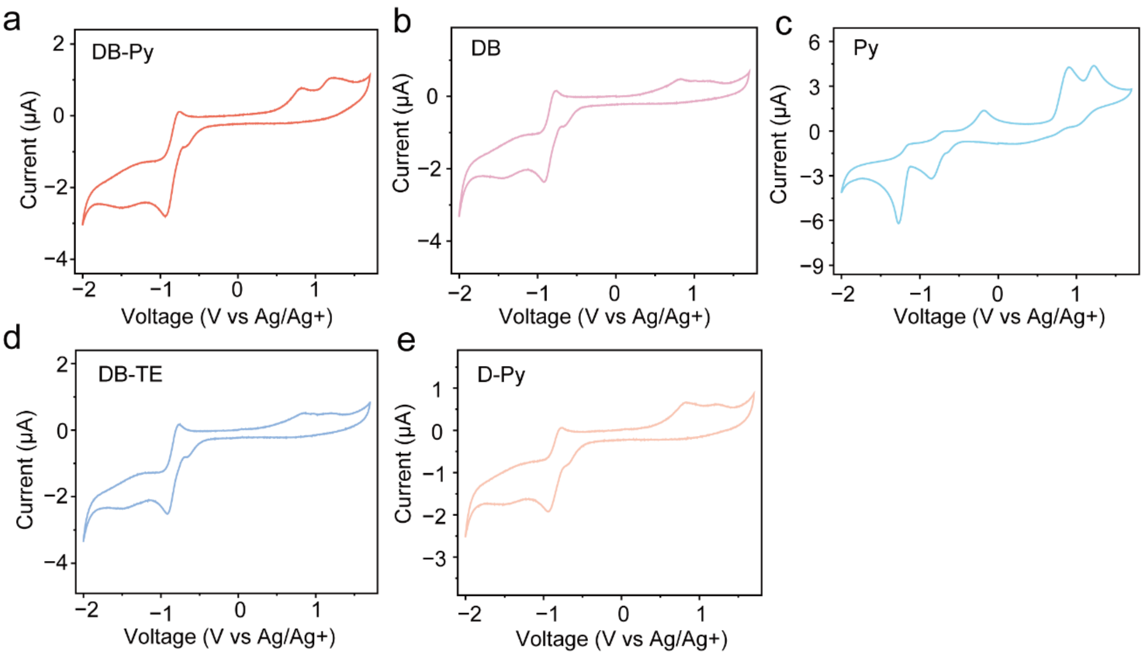

Fig. S24. Cyclic voltammetry of (a) DB-Py, (b) DB, (c) Py, (d) DB-TE and (e) D-Py. The electrochemical cyclic voltammetry measurements were performed using an AUTOLAB electrochemical analyzer. A glassy carbon electrode served as the working electrode, a platinum wire as the counter electrode, and an Ag/AgCl electrode as the reference electrode. The experiments were carried out 10 mM $[Bu_4N]$ $[PF_6]$ in DCM at a scan rate of 20 mV s^{-1} for three cycles, within a potential range of -2 V to 1.8 V.

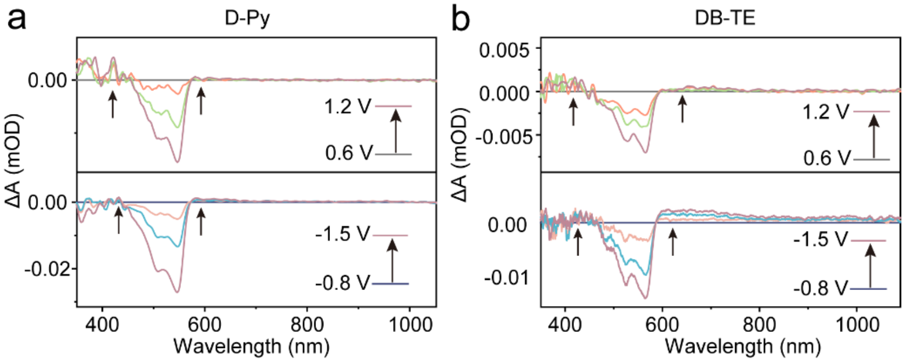

Fig. S25. Spectroelectrochemical analysis of (a) D-Py and (b) DB-TE in DCM under different applied potentials.

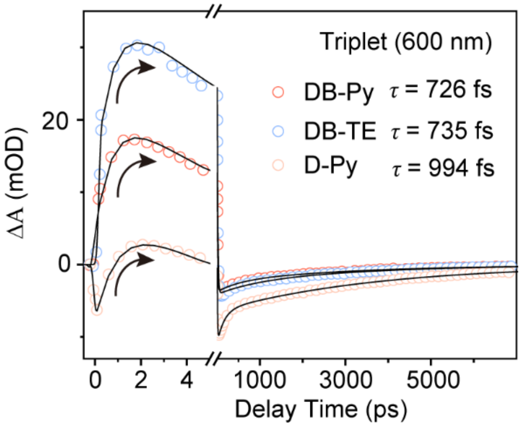

Fig. S26. Kinetic traces and fitting curves for DB-Py, DB-TE and D-Py at the representative wavelength (600 nm) corresponding to the ESA region of triplet-state.

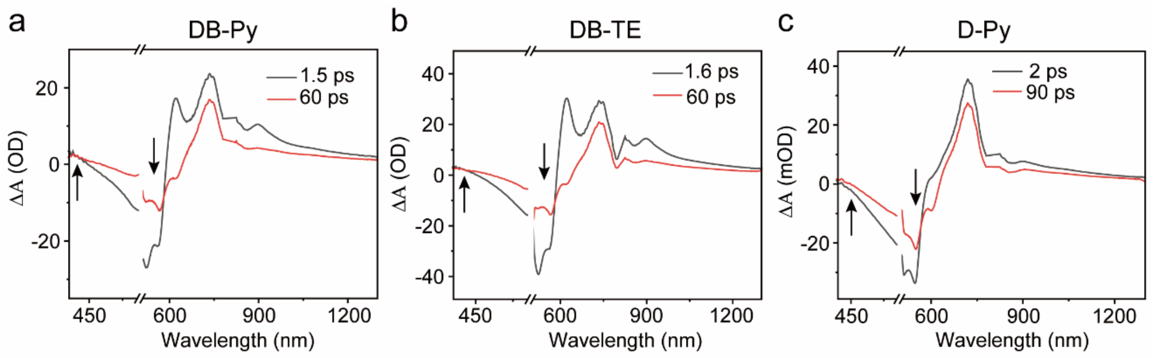

Fig. S27. Extracted fs-TA plots under different delay times of (a) DB-Py, (b) DB-TE and (c) D-Py. The relative radical yields, roughly estimated from the intensity ratio between the 440 nm ESA and 550 nm GSB signals at their maximum intensity, follow the order DB-Py (25.0%) > DB-TE (15.1%) > D-Py (12.9%).

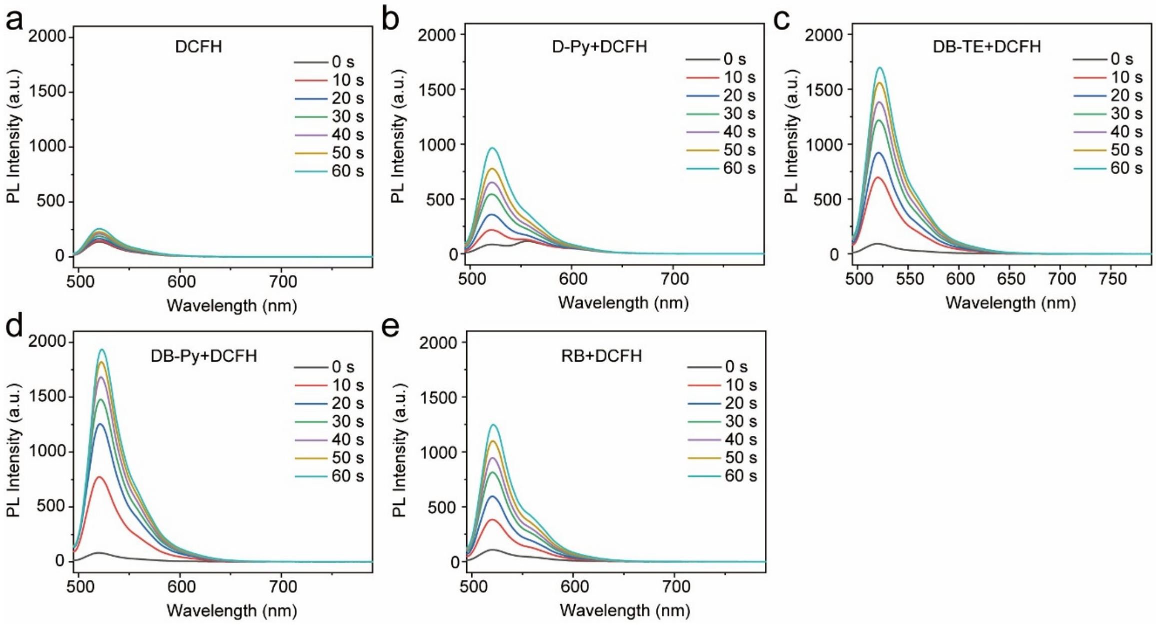

Fig. S28. PL spectra of DCFH after irradiation in the presence of (a) DCFH, (b) D-Py + DCFH, (c) DB-TE + DCFH, (d) DB-Py + DCFH and (e) RB + DCFH. During irradiation, a 495 nm long-pass filter was placed in front of the white-light, and the light intensity was adjusted to 50 mW cm^{-2}.

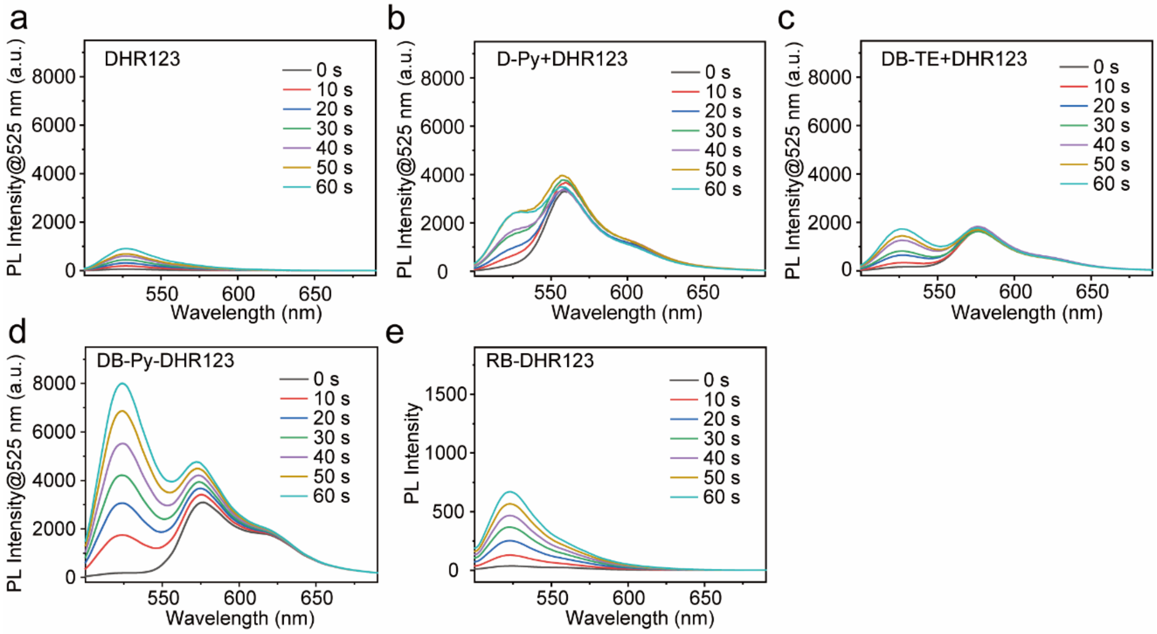

Fig. S29. PL spectra of DHR123 after irradiation in the presence of (a) DHR123, (b) D-Py + DHR123, (c) DB-TE + DHR123, (d) DB-Py + DHR123 and (e) RB + DHR123. During irradiation, a 495 nm long-pass filter was placed in front of the white-light, and the light intensity was adjusted to 50 mW cm^{-2}.

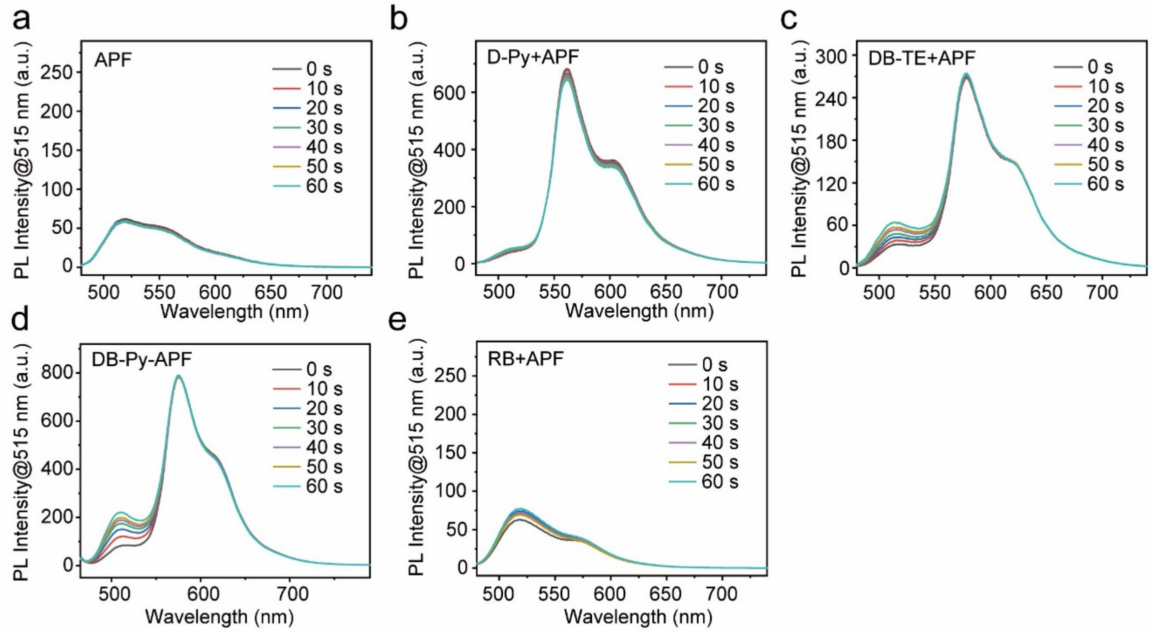

Fig. S30. PL spectra of APF after irradiation in the presence of (a) APF, (b) D-Py + APF, (c) DB-TE + APF, (d) DB-Py + APF and (e) RB + APF. During irradiation, a 495 nm long-pass filter was placed in front of the white-light, and the light intensity was adjusted to 50 mW cm^{-2}.

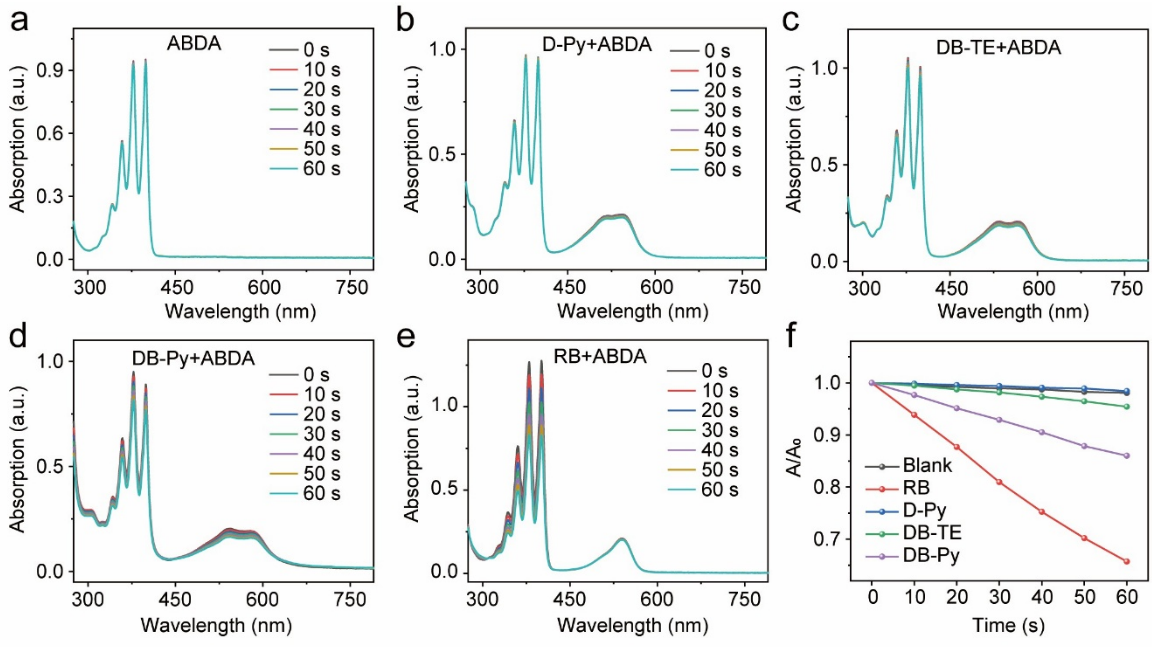

Fig. S31. Generation of $^1O_2$ in various conditions using ABDA indicator. The absorption spectra change over 60 s of (a) ABDA, (b) D-Py + ABDA, (c) DB-TE + ABDA, (d) DB-Py + ABDA and (e) RB + ABDA. During irradiation, a 495 nm long-pass filter was placed in front of the white-light, and the light intensity was adjusted to 50 mW cm^{-2}. (f) ABDA degradation (monitored at 398 nm) induced by different PS under white-light irradiation (50 mW cm^{-2}). The stronger $^1O_2$ production, the more degradation of ABDA.

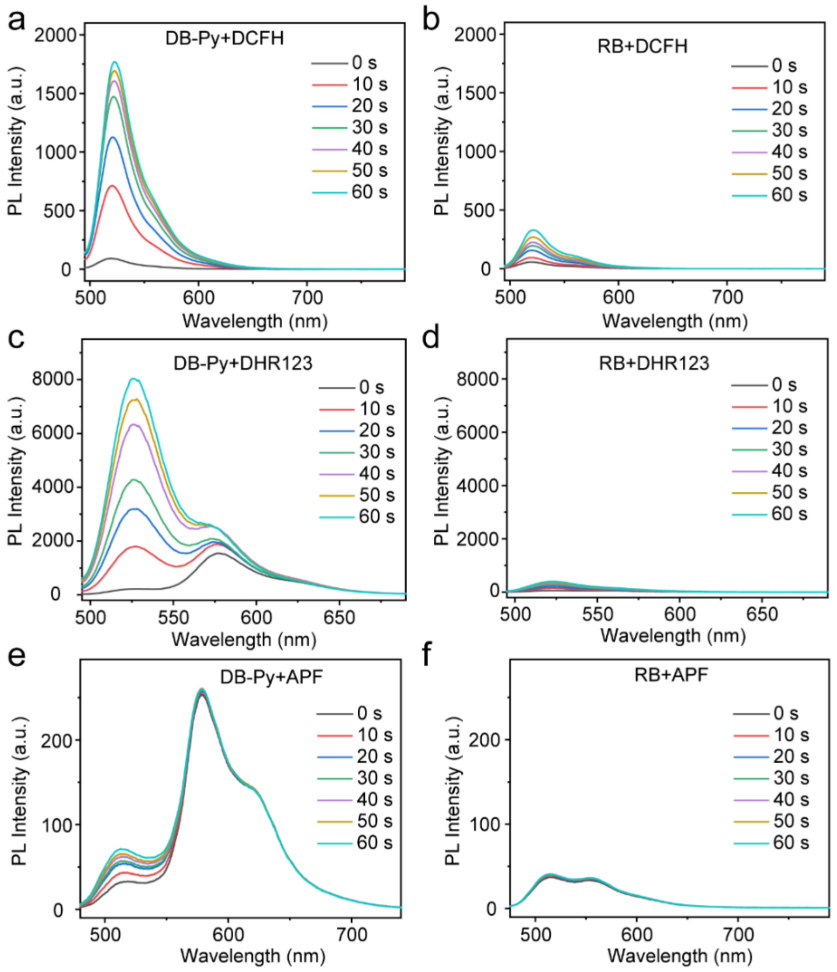

Fig. S32. PL spectra of ROS probes under hypoxic conditions after irradiation in the presence of (a) DB-Py + DCFH, (b) RB + DCFH, (c) DB-Py + DHR123, (d) RB + DHR123 and (e) DB-Py + APF, (f) RB +APF. White light was filtered through a 495 nm long-pass filter, with the intensity adjusted to 50 mW cm^{-2}.

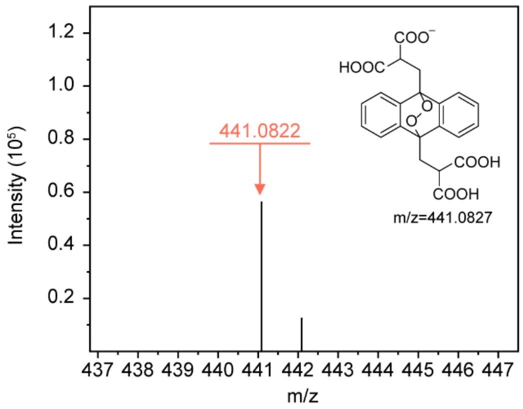

Fig. S33. Isotopic mass spectrometry spectra of photoinduced O_2 production from DB-Py in H_2O solution after 30-min white-light irradiation.

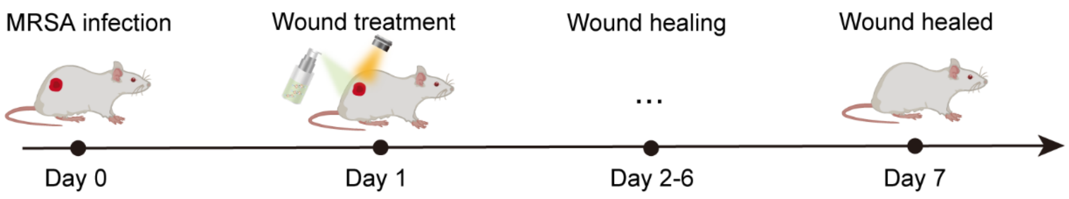

Fig. S34. Illustration of the administration process for *in vivo* antibacterial PDT.

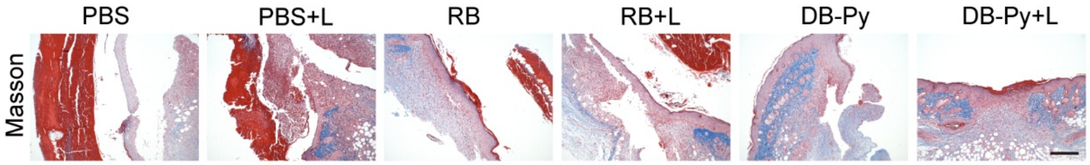

Fig. S35. Masson-stained images of wound tissue after 8 days of treatment (scale bar: 200 μm).

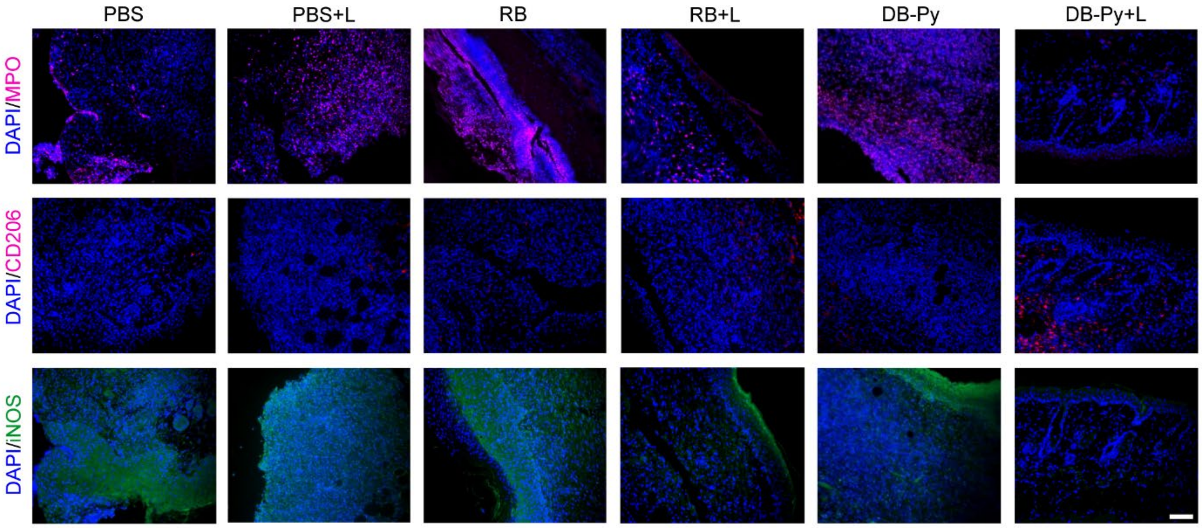

Fig. S36. Immunohistochemical staining of MPO, CD206, and iNOS in skin tissue from different groups (scale bar: 50 μm). Immunofluorescence staining indicates reduced inflammation and a more favorable wound-healing response in the DB-Py-treated group.

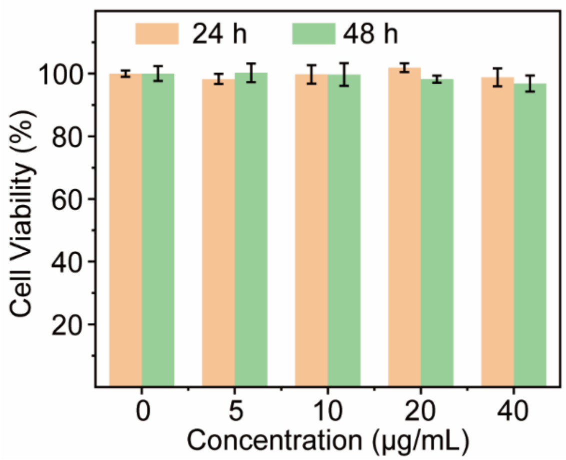

Fig. S37. Cell viability of NIH 3T3 cells after co-incubation with DB-Py extracts for 24 and 48 h. The results show that DB-Py induces no significant cytotoxicity towards NIH 3T3 cells.

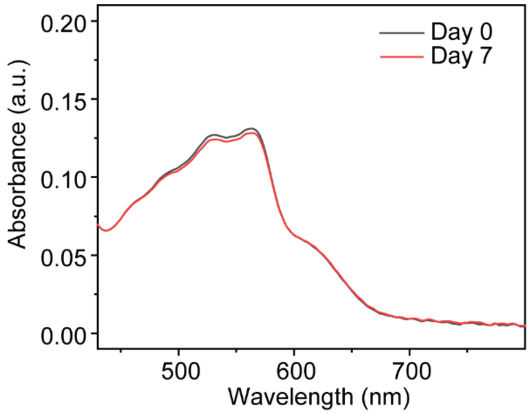

Fig. S38. Absorption spectral changes of DB-Py in 10% FBS solution on days 0 and 7.

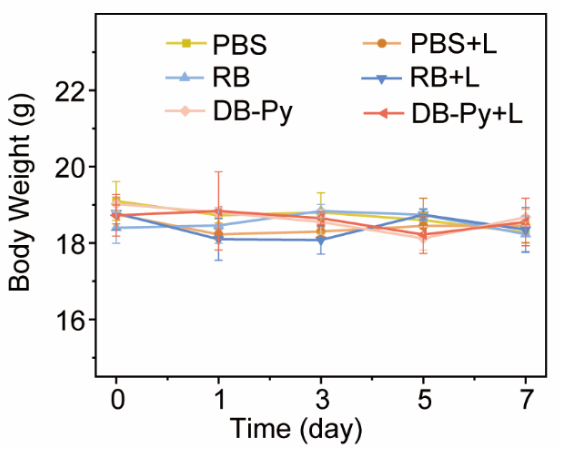

Fig. S39. Body-weight changes of mice in different treatment groups during therapy. No significant body-weight changes were observed in any group during the treatment period, indicating the good biocompatibility (data presented mean ±SD, n = 4).

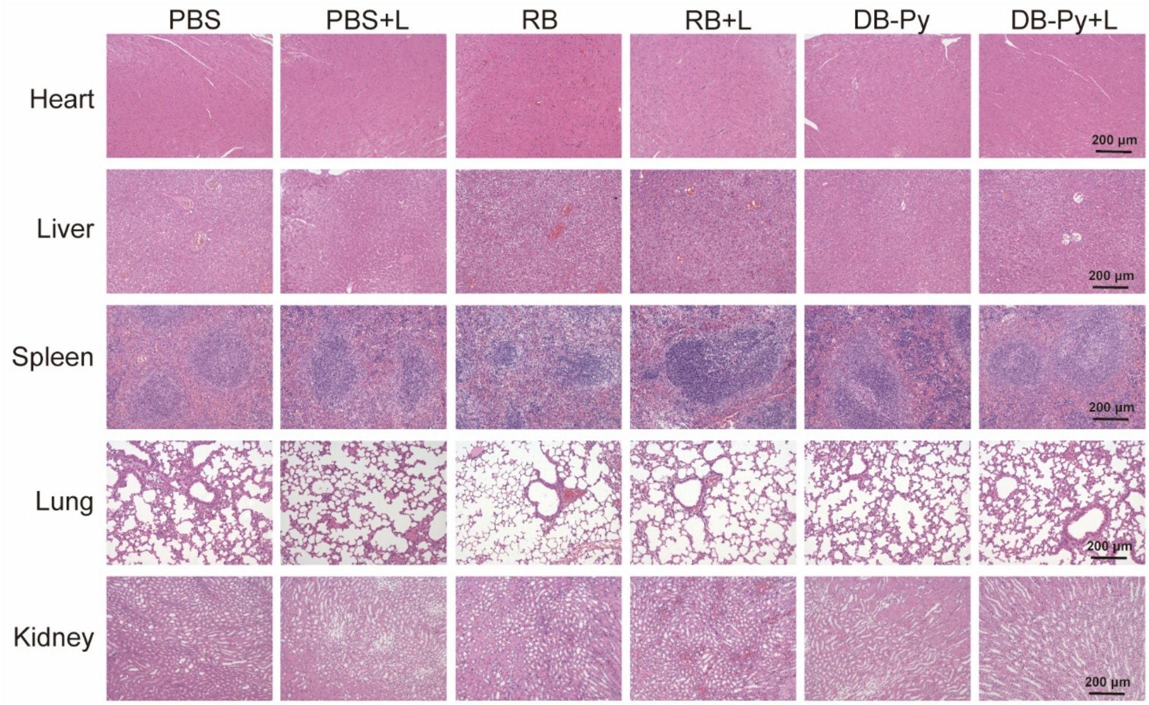

Fig. S40. H&E staining of major organs from mice in different treatment groups. (scale bar: 200 μm). No significant histological abnormalities or tissue damage were observed, indicating the good *in vivo* biosafety of DB-Py.

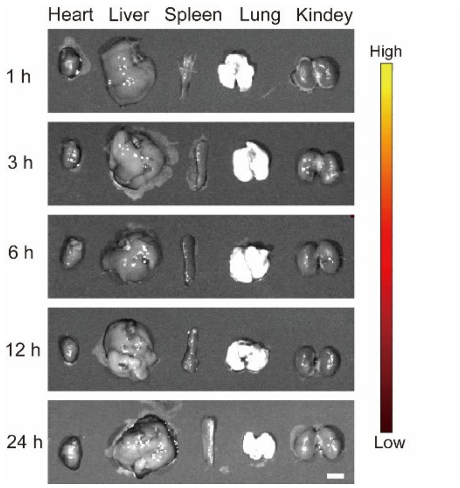

Fig. S41. Fluorescence images of major organs collected from mice at different time points after DB-Py administration. (scale bar: 5 mm)

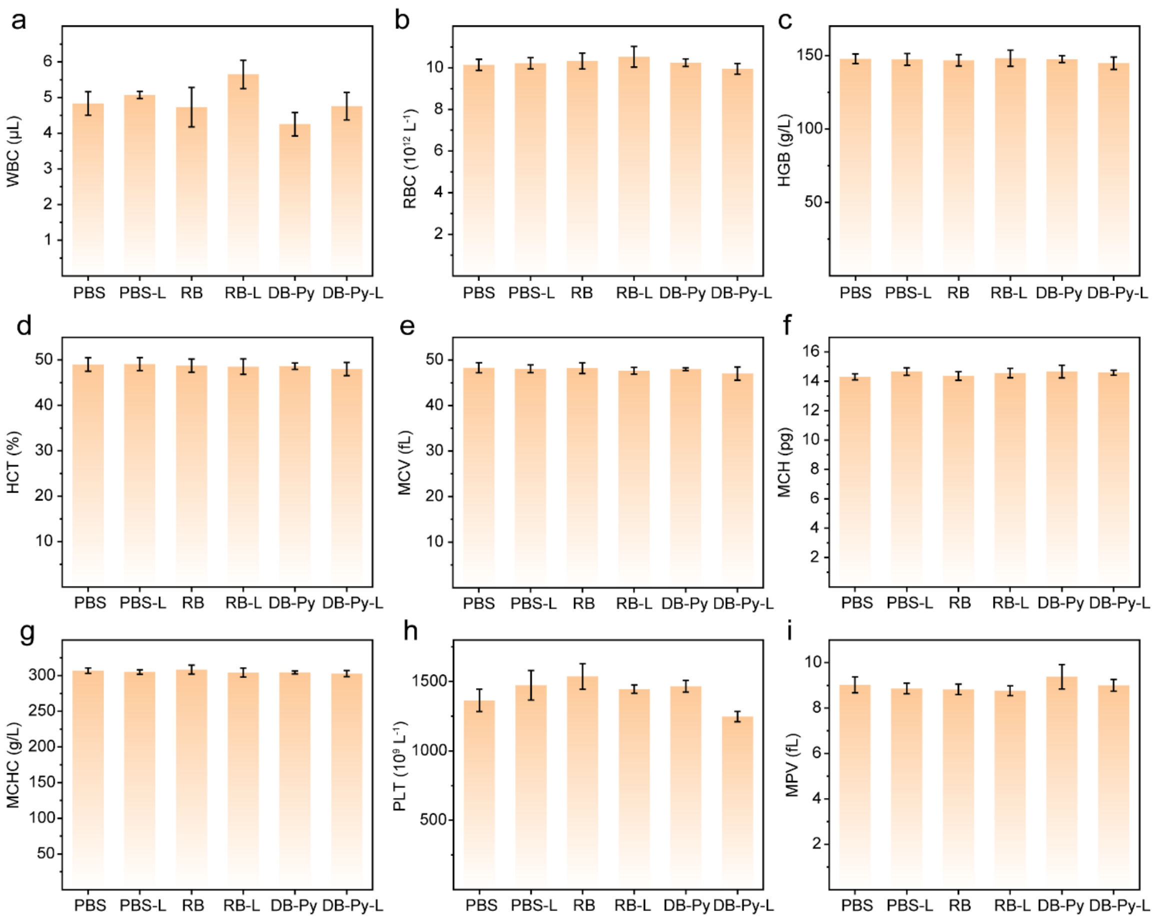

Fig. S42. Results of routine blood tests. (a) White blood cell count (WBC); (b) Red blood cell (RBC); (c) Hemoglobin (HGB); (d) Hematocrit (HCT); (e) Mean corpuscular volume (MCV); (f) Mean corpuscular hemoglobin (MCH); (g) Mean corpuscular hemoglobin concentration (MCHC); (h) Platelet count (PLT); (i) Mean platelet volume (MPV).

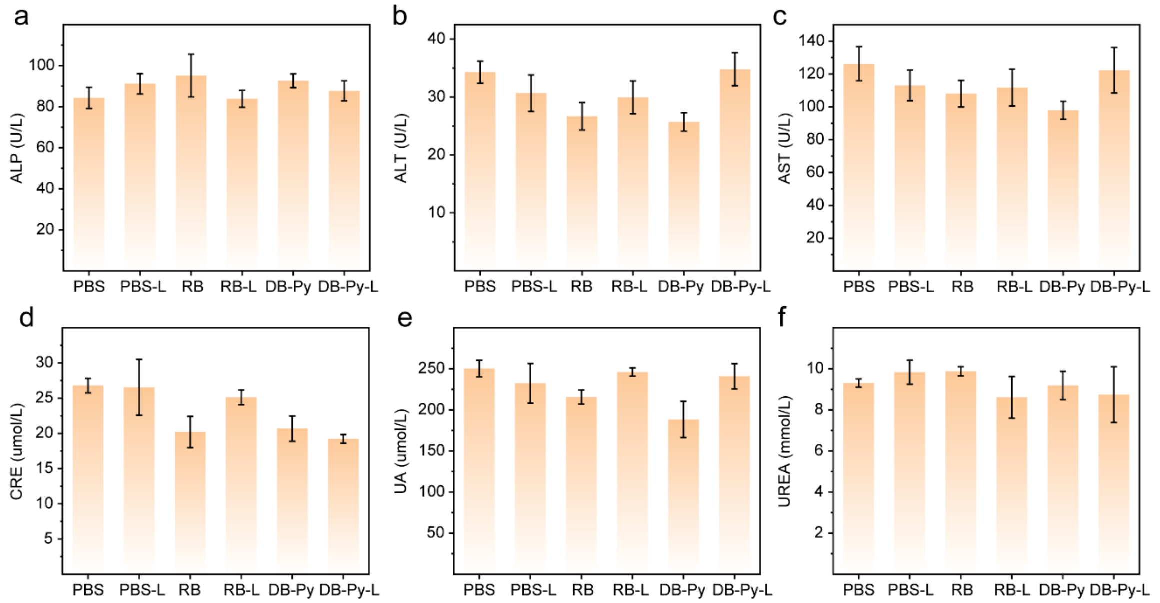

Fig. S43. Results of liver and kidney function tests in mice with different treatments. (a) Alkaline phosphatase (ALP); (b)Alanine aminotransferase (ALT); (c) Aspartate aminotransferase (AST); (d) Creatinine (CRE); (e) Uric acid (UA); (f) Urea (UREA).

Table S1. Photophysical parameters related to excited-state deactivation of the PSs.

PSs	Radiative decay		Nonradiative decay	
	Fluorescence lifetime, ns	PLQY, %	Stokes shift, cm^{-1} (Vibrational relaxation)	Photothermal, °C (Internal conversion)
D-Py	4.1 (560 nm)	31.3	885.4	32.0
DB-TE	3.2 (575 nm)	19.7	811.7	33.6
DB-Py	3.1 (575 nm)	18.2	766.9	31.0

Table S2. The relative electronic energies of the six selected lowest-energy conformations of the DB-Py homodimer, referenced to the most stable conformation (Homodimer 6).

Dimers	Relative energy (kcal/mol, *vs*. Homodimer 6)
Homodimer 1	7.4
Homodimer 2	9.0
Homodimer 3	8.2
Homodimer 4	10.0
Homodimer 5	4.1
**Homodimer 6**	**0**

Table S3. VEA and VIP values (eV) computed in water (iefPCM solvent modal) at B3LYP-D3(BJ)/ma-TZVP(-f) level of theory for DB-Py.

VEA (S_0)	VIP (S_0)	VEA (S_1)	VIP (S_1)	VEA (T_1)	VIP (T_1)	VEA (T_2)	VIP (T_2)
-3.4	5.7	-5.6	3.3	-4.5	4.4	-5.9	3.1

The thermodynamic feasibility of autoionization was assessed by calculating the vertical ionization potential (VIP) and vertical electron affinity (VEA) of the PSs, where a negative sum indicates a favorable reaction[7]. The results show that autoionization-driven fission is theoretically allowed only between a photoexcited triplet-state (T_2) DB-Py molecule and a ground-state (S_0) molecule (Table S2).

SI References

1. Stewart, J. et al. MOPAC. Zenodo 2022, DOI: 10.5281/zenodo.6511959.

2. Lu, T. Molclus program, Version 1.12, http://www.keinsci.com/research/molclus.html

3. Lu, T., & Chen Q. X. Independent gradient model based on Hirshfeld partition: A new method for visual study of interactions in chemical systems, *Journal of Computational Chemistry* **43**, 539-555 (2022).

4. Lu, T. et al. Multiwfn: A multifunctional wavefunction analyzer. *Journal of Computational Chemistry* **33**, 580-592 (2011).

5. Lu, T. A comprehensive electron wavefunction analysis toolbox for chemists, Multiwfn. *Journal of Chemical Physics* **161**, 082503 (2024).

6. Humphrey, W., Dalke, A. & Schulten, K. VMD: visual molecular dynamics. *Journal of Molecular Graphics and Modelling* **14**, 33-38 (1996).

7. He, P. et al. Zwitterionic photosensitizer-assembled nanocluster produces efficient photogenerated radicals via autoionization for superior antibacterial photodynamic therapy. *Advanced Materials* **37**, 2418978 (2025).
